# Supplementary material for: Restoration of retinal regenerative potential of Müller glia by disrupting intercellular Prox1 transfer
Source: Nat Commun. 2025 Mar 25;16:2928. doi: 10.1038/s41467-025-58290-8 (PMC11937340; doi:10.1038/s41467-025-58290-8)
Supplement: Supplementary file 1 — Supplementary Information [file 41467_2025_58290_MOESM1_ESM.pdf]

Supplementary Information for

Restoration of retinal regenerative potential of Müller glia by disrupting intercellular Prox1 transfer

Eun Jung Lee *et al.*

This file includes:

Supplementary Figure 1 – 24
Supplementary Table 1
Supplementary Material 1 and 2

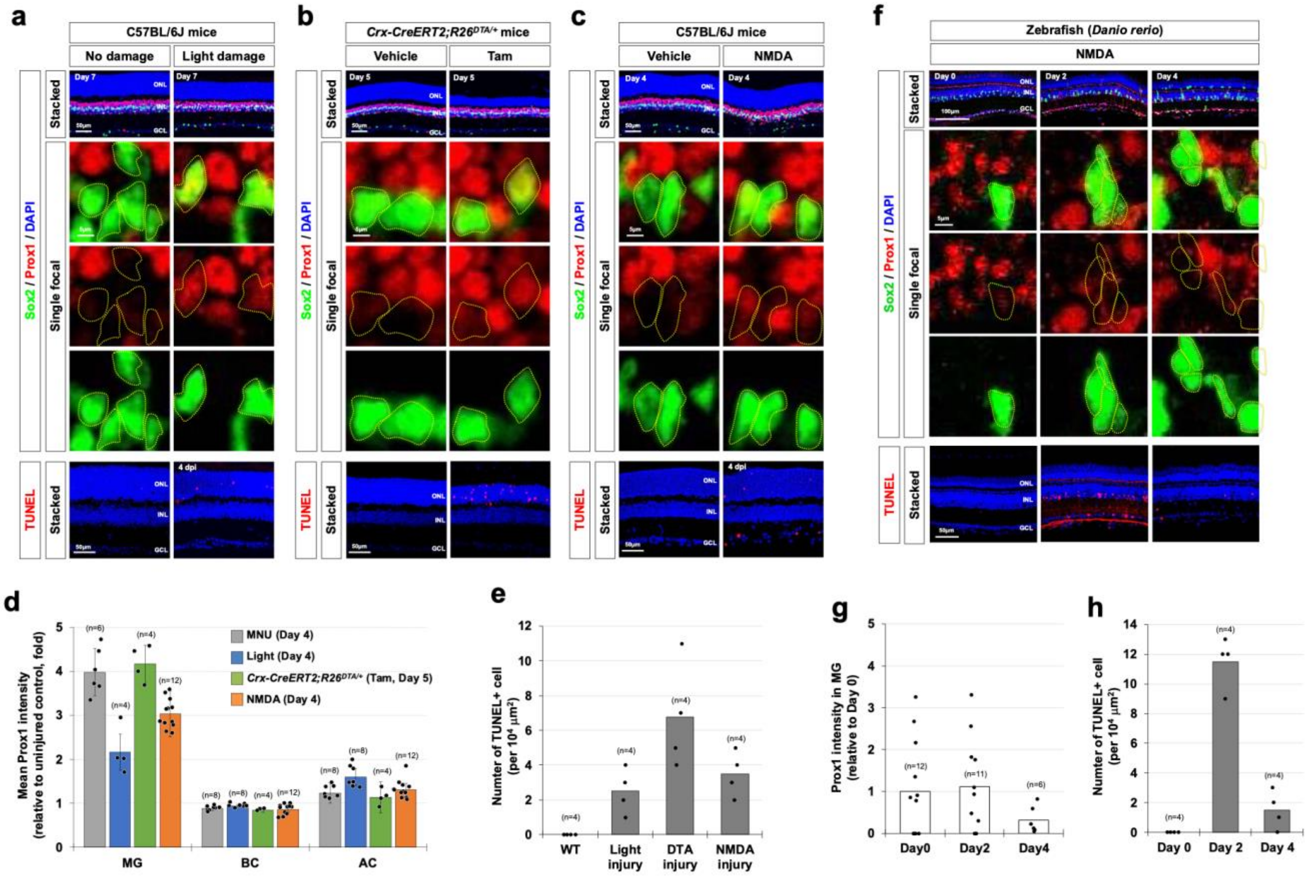

Supplementary Figure 1. Upregulation of Prox1 in the MG of injured mouse retinas. (a) P30 C57BL/6J mice, dark-adapted and with dilated pupils, were exposed to light (100,000 Lux) for 3 hours. (**b**) P30 *R26*$^{DTA/+}$*;Crx-CreERT2* mice were administered Tam (75 mg/kg) to activate CreERT2 , facilitating the deletion of the loxP-STOP-loxP (LSL) cassette to express diphtheria toxin A (DTA) in the photoreceptors. **(c)** P30 C57BL/6J mice were intravitreally injected with 0.5 µl of PBS (vehicle) or PBS containing NMDA (100 mM). After the days indicated in the images, the eyes were isolated for immunostaining to assess Prox1 expression. Sox2-positive MG cell nuclei are outlined by dotted lines. **(d)** Prox1 intensities in MG and ACs were normalized to that of BCs within the same images and their relative intensities to uninjured control mouse retinas are presented in the graph. Numbers of samples analyzed are shown in the graph (data from 3 independent litters). **(e)** The number of TUNEL(+) apoptotic cells per retinal area is presented in the graph. **(f)** Zebrafish retinas, aged 6 months, were injured via intraocular injection of 2 µl NMDA (100 mM). Distribution of Prox1 in these fish retinas was then assessed by immunostaining. **(g)** Prox1 intensities in MG were normalized to that of BCs within the same images and their relative intensities to uninjured control mouse retinas (Day 0) are presented in the graph. Numbers of samples analyzed are shown in the graph (data from 3 independent litters). **(h)** The number of TUNEL(+) apoptotic cells per retinal area is presented in the graph.

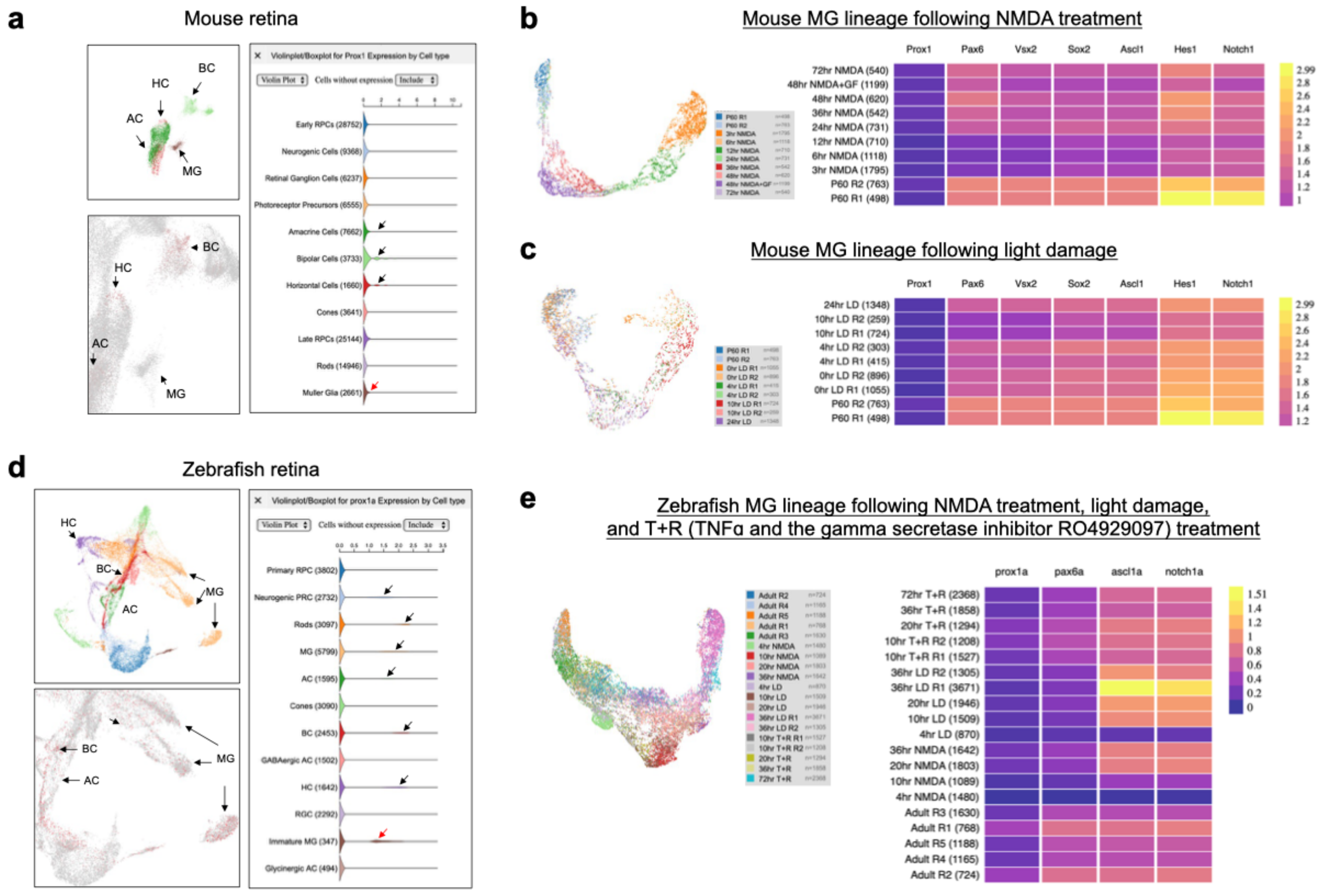

Supplementary Figure 2. Insignificant changes in Prox1 expression in MG of injured mouse and zebrafish retinas. The presence of Prox1-expressing cells in mouse and zebrafish retinas was determined by analyzing single-cell RNA sequencing (scRNA-seq) data available at https://proteinpaint.stjude.org/F/2019.retina.scRNA.html. **(a)** In mouse retina, *Prox1* mRNA was detectable in BC, HC, and AC at significant level (black arrowheads); however, it was barely detectable in MG (red arrowheads). **(b and c)** Analysis of purified MG lineage cells from mouse retinas subjected to NMDA damage (b) or intense light damage (LD, c) revealed a scarce level of *Prox1* mRNA, which did not show significant changes following the injuries. **(d)** In zebrafish retina, *prox1a* mRNA was broadly detectable in neurogenic RPC, rods, MG, AC, BC, HC and immature MG. **(e)** In purified MG lineage cells from zebrafish retinas exposed to NMDA, LD or T+R, *prox1a* mRNA did not exhibit significant changes.

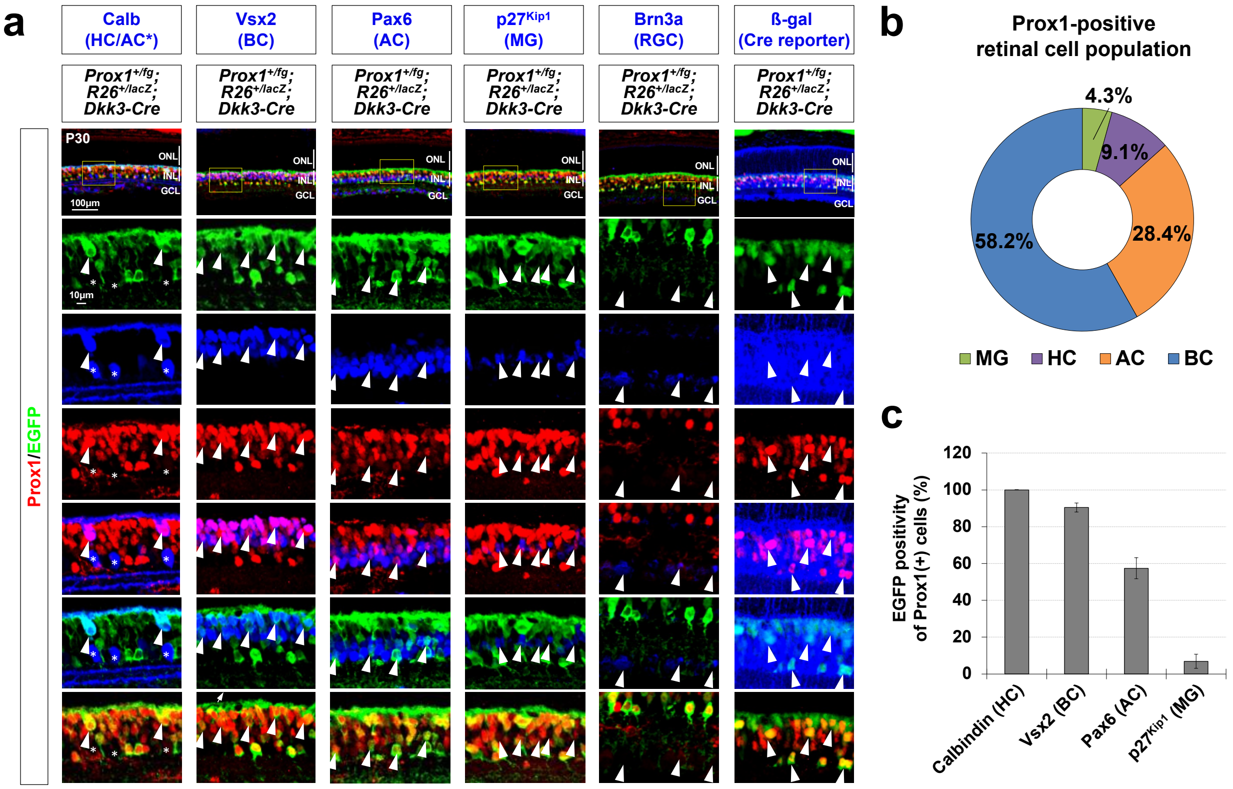

Supplementary Figure 3. *Prox1* gene expression in HC, BC, and AC population in mouse retina. (a) The distribution of Prox1 and EGFP in the P30 *Prox1$^{+/fg}$;Dkk3-Cre* mouse retina was investigated. The identification of Prox1- and EGFP-expressing cells in the retinas was conducted through co-immunostaining with corresponding markers for each retinal cell type. Cells undergoing Cre-dependent DNA recombination were visualized by immunostaining for ß-galactosidase (ß-gal) expressed in the *R26^{lacZ}* locus. Boxed areas in the images in the top row are magnified in the bottom rows. Arrowheads point Prox1(+);Marker(+) cells. (**b**) Numbers of cells expressing corresponding markers in the retinas of P30 *Prox1$^{+/fg}$;R26$^{+/lacZ}$;Dkk3-Cre* mice counted and their ratio to total Prox1-expressing cell number was presented in the graph. **(c)** EGFP positivity of each Prox1-expressing retinal cell subset. Error bars represent SEM (n=6, 3 independent litters).

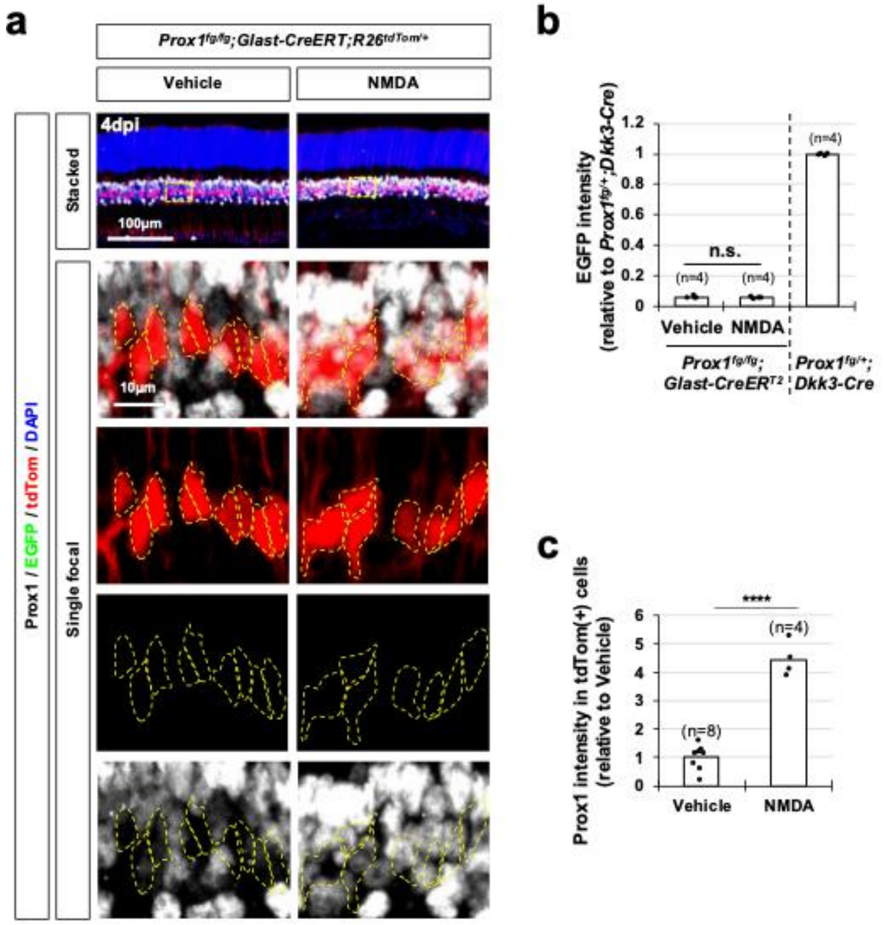

Supplementary Figure 4. NMDA-induced accumulation of Prox1 protein in MG of *Prox1-cko* mice. (a) *Prox1$^{fg/fg}$;Glast-CreERT;R26$^{+/tdTom}$* (*Prox1-cko*) littermate mice were administered Tam at P21 and P22 to activate CreERT recombinase, leading to the deletion of Prox1 in the MG population prior to NMDA injection at P26. The endogenous expression of Prox1 in MG was indirectly determined by assessing EGFP expression in the *Prox1* gene locus. Boxed areas in top row are magnified in the next rows. The tdTom-positive cell bodies are outlined by dotted lines. **(b)** EGFP intensity of tdTom-positive cell body area was measured and the relative intensity to EGFP intensity of *Prox1$^{fg/fg}$;Dkk3-Cre* mouse retina was presented in the graph. **(c)** The graph illustrates Prox1 intensity in MG relative to Prox1 intensity in MG of Vehicle-treated littermate mouse retina. Numbers of samples analyzed are shown in the graphs (data from 2 independent litters). P-values were calculated using one-sided Student's t-test (**** $p<0.001$; n.s. >0.05).

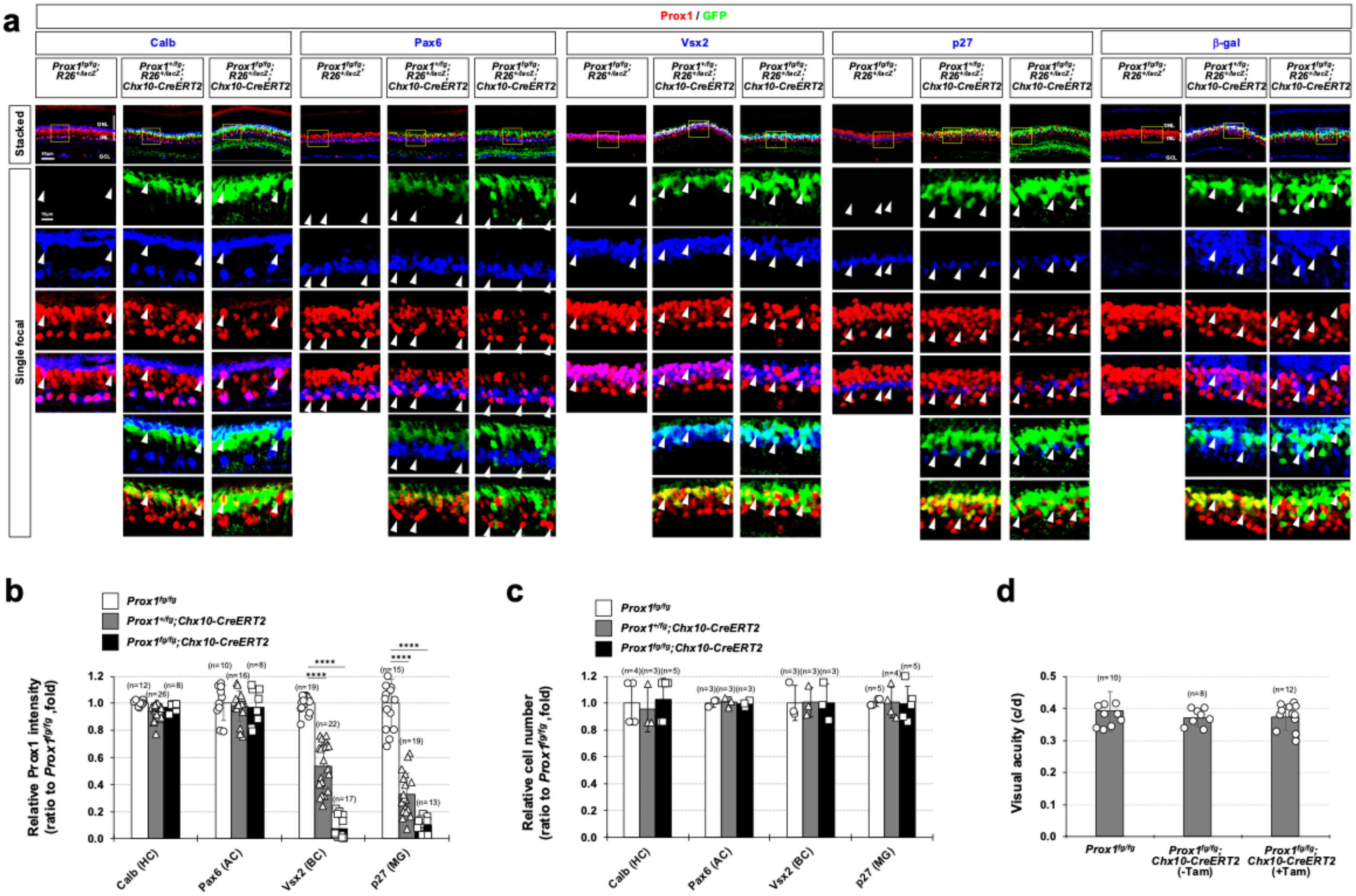

Supplementary Figure 5. Deletion of *Prox1* in the BC population of *Prox1*$^{fg/fg}$*;Chx10-CreERT2* mouse retinas. (a) Mice with the indicated genotypes were injected with Tam at P21, P22, and P23 to activate Chx10-CreERT2 recombinase, which is selectively expressed in BC population. The CreERT2-dependent expression of EGFP in the *Prox1*^{fg} knock-in allele was assessed by confocal microscopy of eye sections from P40 mice. Cells underwent Cre-dependent DNA recombination were visualized by immunostaining for ß-gal Cre reporter. Boxed areas in the images in the top row are magnified in the bottom rows. Arrowheads point Prox1;Marker(+) cells. **(b)** The graph illustrates Prox1 intensities in HC, AC, BC, and MG relative to those in *Prox1*^{fg} mice. **(c)** Number of cells expressing corresponding markers in the mouse retinas were counted and their ratio to the number in the retinas of P30 *Prox1*$^{fg/fg}$ littermates are presented. **(d)** Visual acuities of the mice were measured as described in Methods. Numbers of samples analyzed are shown in the graphs (data from 4 independent litters). P-values were calculated using one-sided Student's t-test (* p<0.05; ** p<0.01; *** p<0.005).

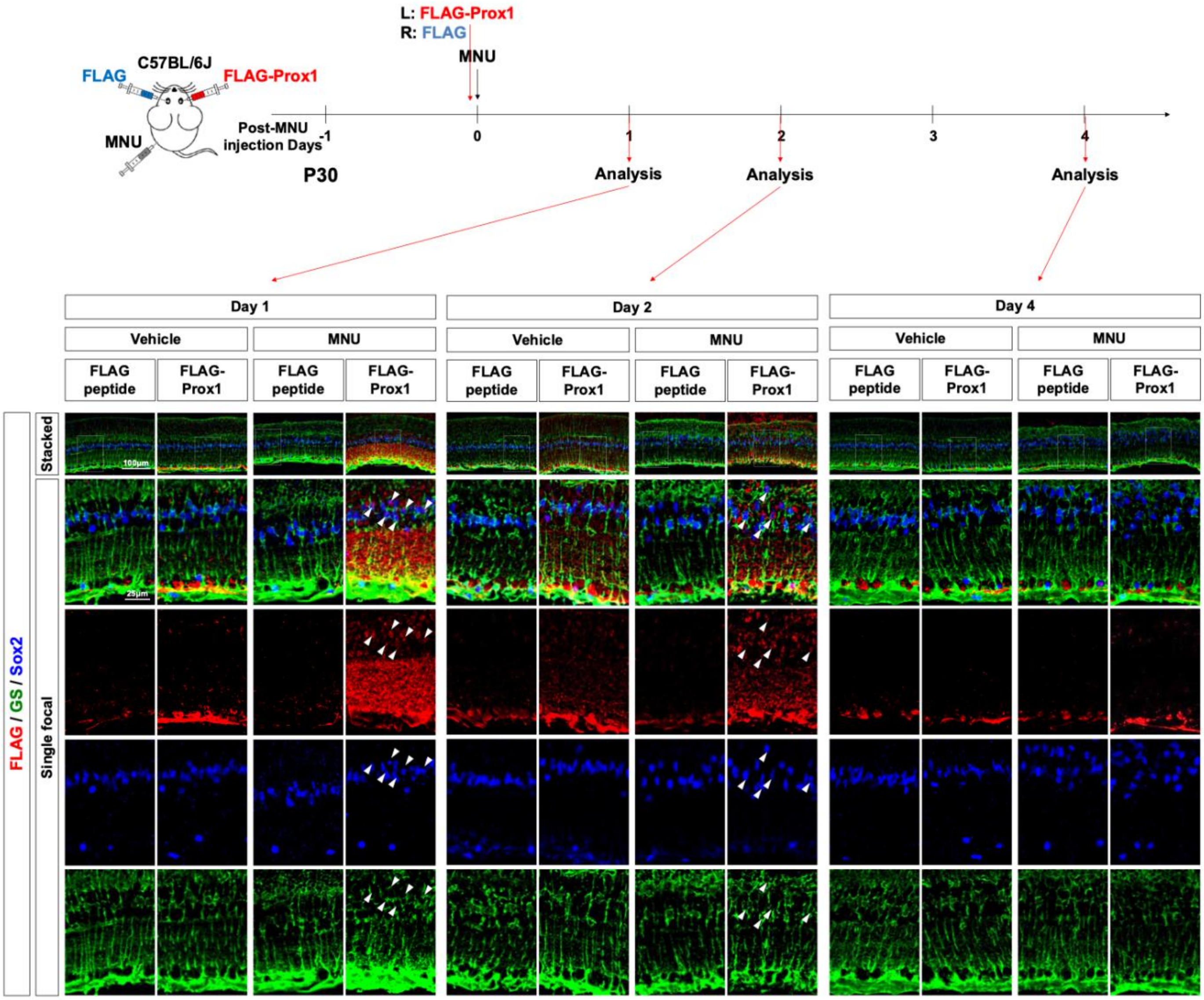

Supplementary Figure 6. Delivery of Prox1 protein to the injured mouse retina. Equal volumes (1 μl) of FLAG peptide (10 ng/μl in PBS; administered to left eyes) and recombinant FLAG-Prox1 protein (20 ng/μl in PBS; administered to right eyes) were injected into the intravitreal spaces of P30 mouse eyes. L, left eyes; R, right eyes. This injection occurred 1 hour after intraperitoneal administration of vehicle (PBS containing 0.05% acetic acid) or MNU (60 mg/kg). The mouse eyes were collected at 1 day (Day 1; P31), 2 days (Day 2; P32), or 4 days (Day 4; P34) post-protein injection, and the presence of the injected FLAG-Prox1 proteins in the mouse retinas was evaluated by immunostaining using an anti-FLAG antibody (red). Penetration of the proteins into MG cells was identified through co-immunostaining with the anti-FLAG antibody and antibodies against MG cell markers, GS and Sox2. Arrowheads point FLAG-positive;Sox2-positive MG nuclei.

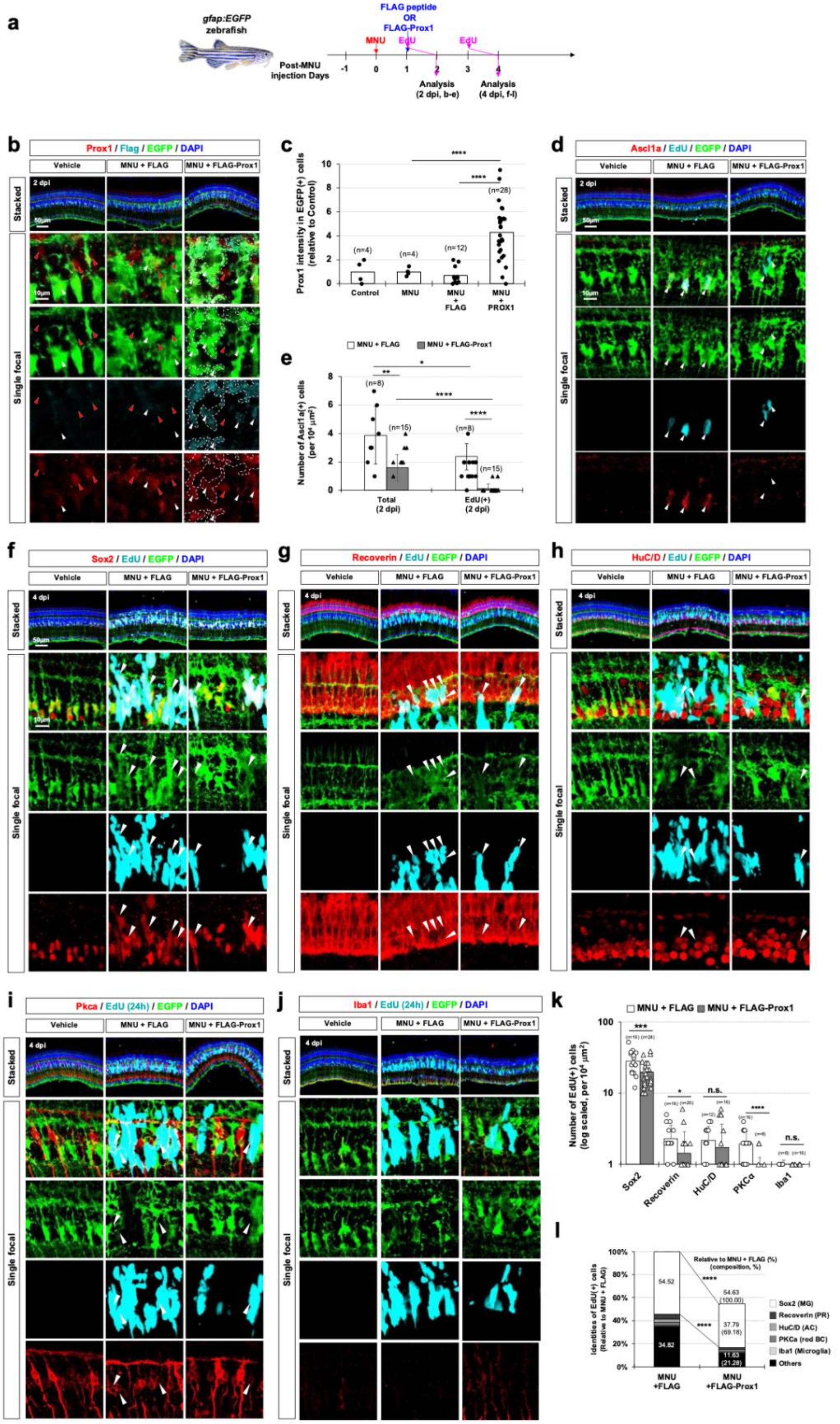
a
gfap:EGFP zebrafish
FLAG peptide OR FLAG-Prox1
MNU
EdU
Post-MNU injection Days
Analysis (2 dpi, b-e)
Analysis (4 dpi, f-l)
b
Prox1 / Flag / EGFP / DAPI
Vehicle
MNU + FLAG
MNU + FLAG-Prox1
Stacked
Single focal
2 dpi
c
Prox1 intensity in EGFP(+) cells (relative to Control)
Control
MNU
MNU + FLAG
MNU + PROX1
d
Ascl1a / EdU / EGFP / DAPI
e
MNU + FLAG
MNU + FLAG-Prox1
Number of Ascl1a(+) cells (per 10⁴ μm²)
Total (2 dpi)
EdU(+) (2 dpi)
f
Sox2 / EdU / EGFP / DAPI
4 dpi
g
Recoverin / EdU / EGFP / DAPI
h
HuC/D / EdU / EGFP / DAPI
i
Pkca / EdU (24h) / EGFP / DAPI
j
Iba1 / EdU (24h) / EGFP / DAPI
k
Number of EdU(+) cells (log scaled, per 10⁴ μm²)
Sox2
Recoverin
HuC/D
PKCα
Iba1
n.s.
l
Identities of EdU(+) cells (Relative to MNU + FLAG)
Relative to MNU + FLAG (%) (composition, %)
Sox2 (MG)
Recoverin (PR)
HuC/D (AC)
PKCa (rod BC)
Iba1 (Microglia)
Others
MNU +FLAG
MNU +FLAG-Prox1

Supplementary Figure 7. Exogenous Prox1 suppresses MG proliferation in the injured zebrafish retina. (a) FLAG peptides or recombinant FLAG-Prox1 proteins were injected into the intravitreal space of MNU-injured *gfap-EGFP* zebrafish eyes, and proliferating cells were labeled with EdU as it is indicated. **(b)** Prox1 distribution in the zebrafish retinas was examined by immunostaining with a rabbit anti-Prox1 antibody, and FLAG-Prox1 was detected by immunostaining with a mouse anti-FLAG antibody. White arrowheads indicate Prox1 in EGFP-positive MG, while red arrowheads point Prox1 in EGFP-negative retinal cells. The FLAG immunostaining signals are outlined by dotted lines. **(c)** The graph illustrates Prox1 intensity in MG relative to other retinal neurons within the same image. **(d)** MGPC identities of EdU-labeled cells in the retinas were determined by co-staining with the cell type-specific marker, Ascl1. White arrowheads point Ascl1;EdU-positive cells. **(e)** The number of Ascl1-positive cells in the zebrafish retinas is presented. **(f – j)** Additionally, the identities of EdU-labeled newborn cells in the zebrafish retinas were determined by co-staining with retinal cell type-specific markers, including Sox2 for MG or MGPCs (f); Recoverin for PR (g); HuC/D for AC (h); Pkcα for BC (i); and Iba1 for microglia (j). Arrowheads indicate EdU-labeled marker-positive cells. **(k)** The number of EdU-labeled cells expressing the markers are shown in the graph. **(l)** Cell composition of EdU-labeled cells in MNU-injured fish retinas is depicted in the graph. For MNU+FLAG-Prox1 samples, the values the relative to the numbers of cells in MNU+FLAG samples are presented, together with cell composition in the retina in the brackets. Others represent EdU(+) cells, which do not express the listed markers, could include retinal cells, including cone photoreceptors and cone BCs, and non-retinal origins, astrocytes and endothelial cells. Numbers of samples analyzed are shown in the graphs (data from 3 independent litters). P-values were calculated using one-sided Student's t-test (*p < 0.05; ** p < 0.01; *** p < 0.005; ****, p < 0.001).

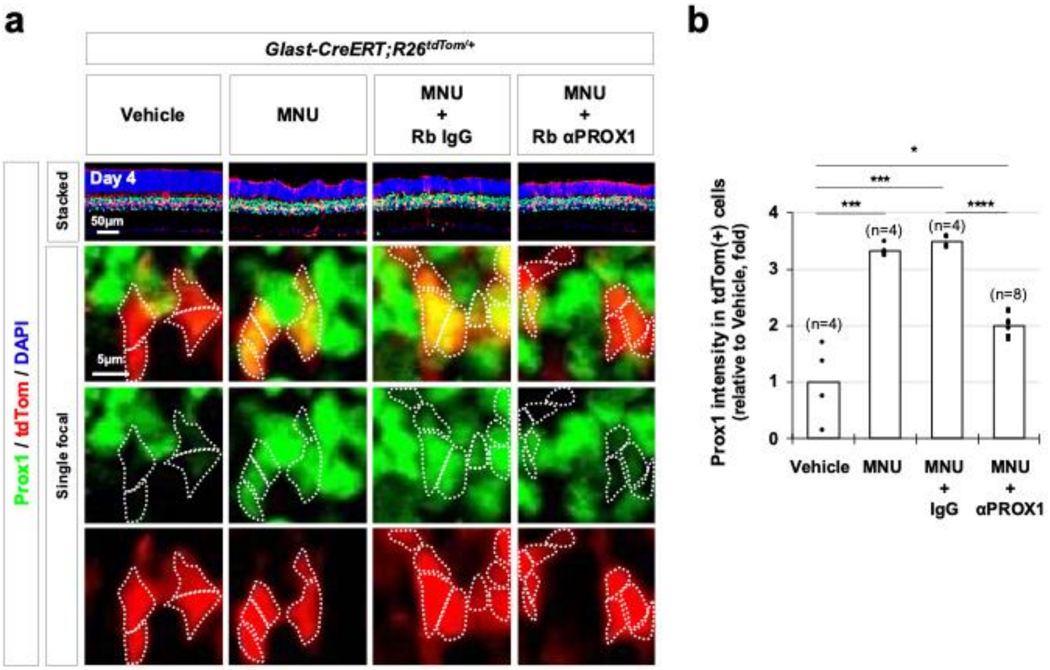

Supplementary Figure 8. Decrease of Prox1 in MG of MNU-injured mouse retinas by intravitreal injection of anti-Prox1 antibody. (**a**) Tam was injected into the peritoneal space of *Glast-CreER;R26*$^{tdTom/+}$ mice at P14 to activate CreER in MG in the mouse retinas. MNU was injected into the peritoneal space of the mice at P30 to degenerate PRs in the retinas. Concomitantly, rabbit immunoglobulin G_1 (Rb IgG) or rabbit anti-Prox1 polyclonal antibody (Rb αProx1) was injected to the intravitreal space of the MNU-injected mice. The eye sections were prepared from the mice at 4 days after the MNU injury. Distribution of Prox1 in the mouse retinal sections was visualized by immunostaining. The activation of CreERT in MG was determined by detecting Cre reporter tdTom. Nuclei of retinal cells in the sections were visualized by DAPI staining. tdTom-positive MG cell bodies are outlined by dotted lines. (**b**) Prox1 intensities of the tdTom(+) MG cells relative to those in Vehicle-injected control retina are plotted. Numbers of samples analyzed are shown in the graph (data from 3 independent litters). P-values were calculated using one-sided Student's t-test (*p < 0.05; ** p < 0.01; *** p < 0.005; ****, p < 0.001). *, p<0.05; ****, p<0.001; n.s., not significant).

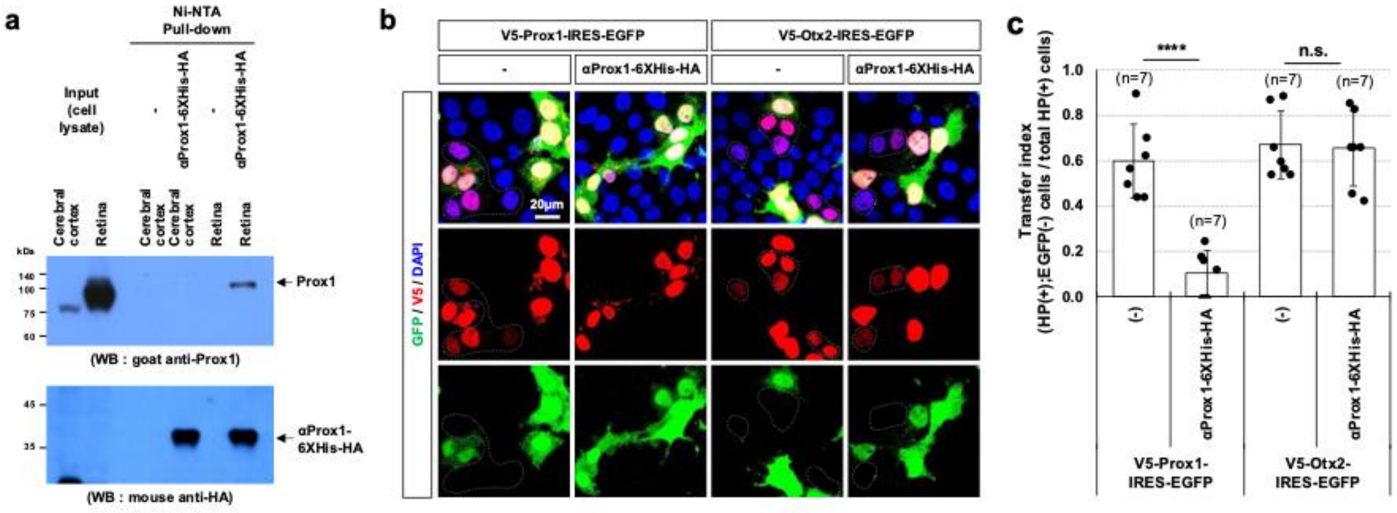

Supplementary Figure 9. Identification of the neutralizing activity of αProx1 towards intercellular Prox1 transfer. (a) The cDNA of anti-Prox1 single-chain variable fragment (αProx1) was cloned into the M13 phagemid to express αProx1-6His-HA in Escherichia coli (E. coli), and the protein was purified using a Ni-NTA column, which captured its 6His tag of the antibody protein (see details in Methods). The αProx1-6His-HA protein (1 μg) was added to cell lysates (containing 1 mg protein) from the adult mouse cerebral cortex or retina and pulled down using the Ni-NTA column. Prox1, bound and co-purified with αProx1-6His-HA, was then detected by Western blot (WB) with goat anti-Prox1 antibody. The αProx1-6His-HA proteins in each lane were also detected by WB with mouse anti-HA antibody. **(b)** The αProx1-6His-HA protein was added to the growth medium of HeLa cells (25 μg/ml), which were transfected with pCAGIG-V5-Prox1 or pCAGIG-V5-Otx2. The cells expressing V5-Prox1 or V5-Otx2 with or without co-transcribed EGFP were identified by co-immunostaining with mouse anti-V5 and chicken anti-GFP antibodies. The cells surrounded by dotted lines contain the homeoproteins (HPs; i.e., Prox1 and Otx2) without EGFP, suggesting that they received the HP from HP;EGFP double-positive neighboring cells. **(c)** The transfer index, which is the ratio of HP(+);EGFP(-) cells among total HP(+) cells, is depicted in the graph. Numbers of samples analyzed are shown in the graph (data from 6 independent experimental batches). P-values were calculated using one-sided Student's t-test (n.s., not significant; **** $p<0.001$).

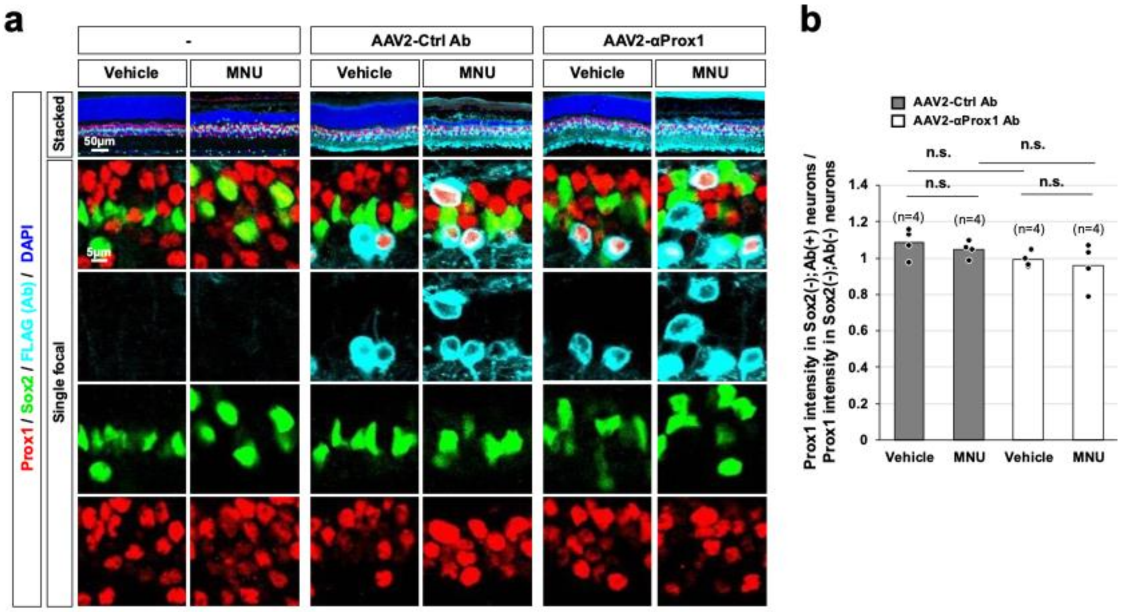

c

Antibody expressing cells		
	Ctrl Ab	Anti-PROX1
RGC	46.81%	41.43%
Lower 50% of INL (AC)	40.43%	49.29%
Upper 50% of INL (BC)	0.00%	0.00%
MG	0.00%	0.00%
HC	11.35%	8.57%
ONL	1.42%	0.71%
Others	0.00%	0.00%
Total	100%	100%

Supplementary Figure 10. Identification of AAV-infected retinal cell populations. (a) P30 C57BL/6J mice were intravitreally injected with AAV2-Ctrl Ab or AAV2-aProx1. At 14 days post-infection, the mice were treated with MNU, and retinal samples were collected 4 days post-injection. AAV-infected cells were identified by immunostaining for the FLAG-tagged antibodies. **(b)** Changes in Prox1 levels in FLAG-positive retinal neurons were quantified and are presented in the graph. **(c)** The retinal composition of FLAG-tagged antibody-expressing cells is summarized in the table. Numbers of samples analyzed are shown in the graph (data from 6 independent experimental batches). P-values were calculated using one-sided Student's t-test (n.s., not significant).

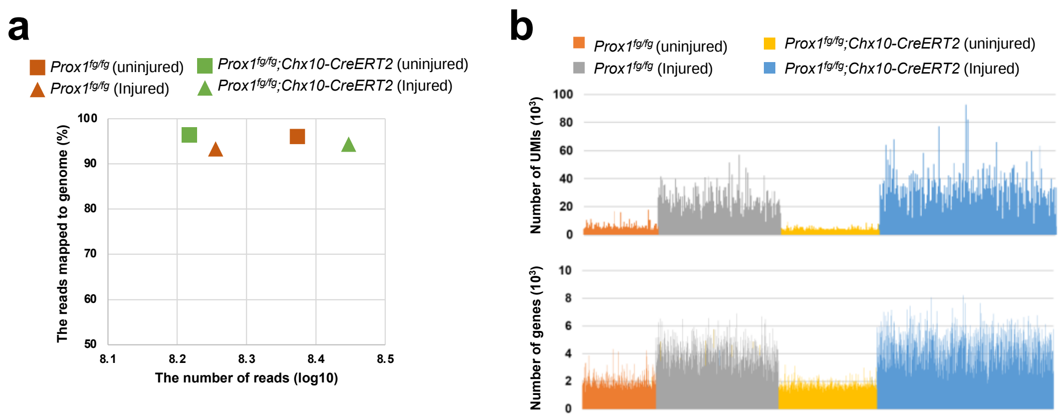

Supplementary Figure 11. Quality of single-cell RNA-seq data. (a) Average number of reads from mouse retina cells with the indicated genotypes and those mapped to genome are presented in the graph. **(b)** Unique molecular identifiers (UMIs) and number of genes in each cell are depicted in the graphs.

a

Cluster #		0	1	2	3	4	5	6	7	8	9	10	11	12	13	14	15	16	17	18	19	20	21	22	23	24	25	26	27	Sum
Prox1$^{fg/fg}$ (Vehicle)	Frequency (%)	56.4	0.4	2.8	3.6	3.1	3.0	2.1	0.1	0.1	1.0	0.1	8.0	2.0	1.1	0.8	1.6	2.7	1.0	1.8	3.0	0.0	0.5	3.1	0.9	0.8	0.0	0.0	0.1	100
Prox1$^{fg/fg}$ (MNU)		2.5	23.6	6.0	9.0	6.3	4.8	5.0	4.9	5.1	4.1	7.1	2.0	3.3	3.6	3.8	2.2	1.6	1.7	0.0	0.2	0.1	1.4	0.1	0.1	0.4	0.4	0.4	0.1	100
Prox1$^{fg/fg}$;*Chx10-CreERT2* (Vehicle)		64.2	0.4	5.7	3.4	1.6	3.2	1.6	0.3	0.0	0.9	0.0	2.6	1.3	1.3	0.3	1.4	0.6	0.7	4.7	2.4	0.0	0.3	2.0	0.4	0.4	0.0	0.0	0.1	100
Prox1$^{fg/fg}$;*Chx10-CreERT2* (MNU)		2.1	20.2	11.6	7.4	6.0	5.0	4.4	5.9	5.9	5.6	4.2	2.9	4.4	2.1	1.7	1.6	1.2	1.8	0.0	0.2	2.7	1.2	0.1	0.8	0.1	0.4	0.3	0.1	100

b

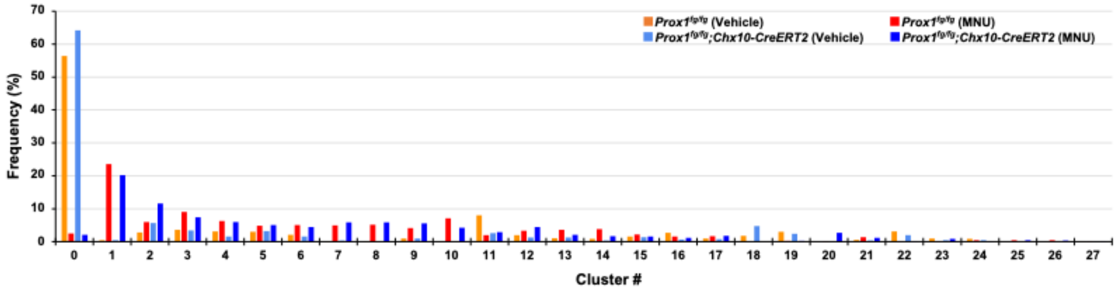

c

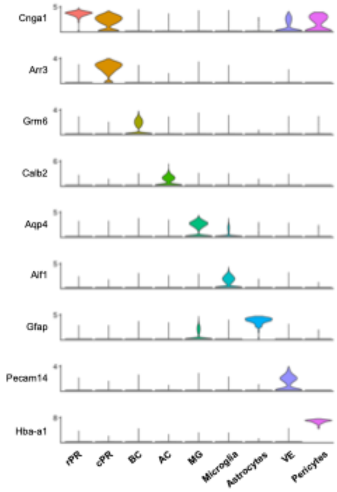

d

Cell types (Cluster #)		rPR (0, 18, 22, 25)	cPR (19)	BC (2, 5, 6, 8, 12,13, 15, 17, 21)	AC (11, 14)	MG (1, 3, 7)	RPC (20)	Microglia (4, 9, 10, 2 1)	Astrocytes (26)	VE (16)	Pericytes (24)	Fibroblast (27)	Total
Prox1$^{fg/fg}$ (Vehicle)	Frequency (%)	61.3	3	14.2	8.8	4.1	0	5.1	0	2.7	0.8	0.1	100
Prox1$^{fg/fg}$ (MNU)		3	0.2	33.1	5.8	37.5	0.1	17.6	0.4	1.6	0.4	0.1	100
Prox1$^{fg/fg}$; *Chx10-CreERT2* (Vehicle)		70.9	2.4	15.5	2.9	4.1	0	2.9	0	0.6	0.4	0.1	100
Prox1$^{fg/fg}$; *Chx10-CreERT2* (MNU)		2.6	0.2	38	4.6	33.5	2.7	16.6	0.3	1.2	0.1	0.1	100

e

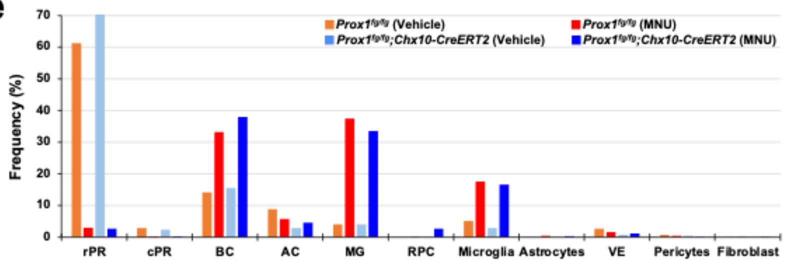

Supplementary Figure 12. scRNA-seq analysis of the cells in MNU-injured mouse retinas. (**a**, **b**) Frequency of each cluster in the mouse retinal cell population. (**c**) Violin plots of the expression of cell type-specific markers identified in previous studies. The cell type of each cluster was defined by these cell type-specific markers. (**d**, **e**) Frequency of each cell type in the mouse retinal cell population.

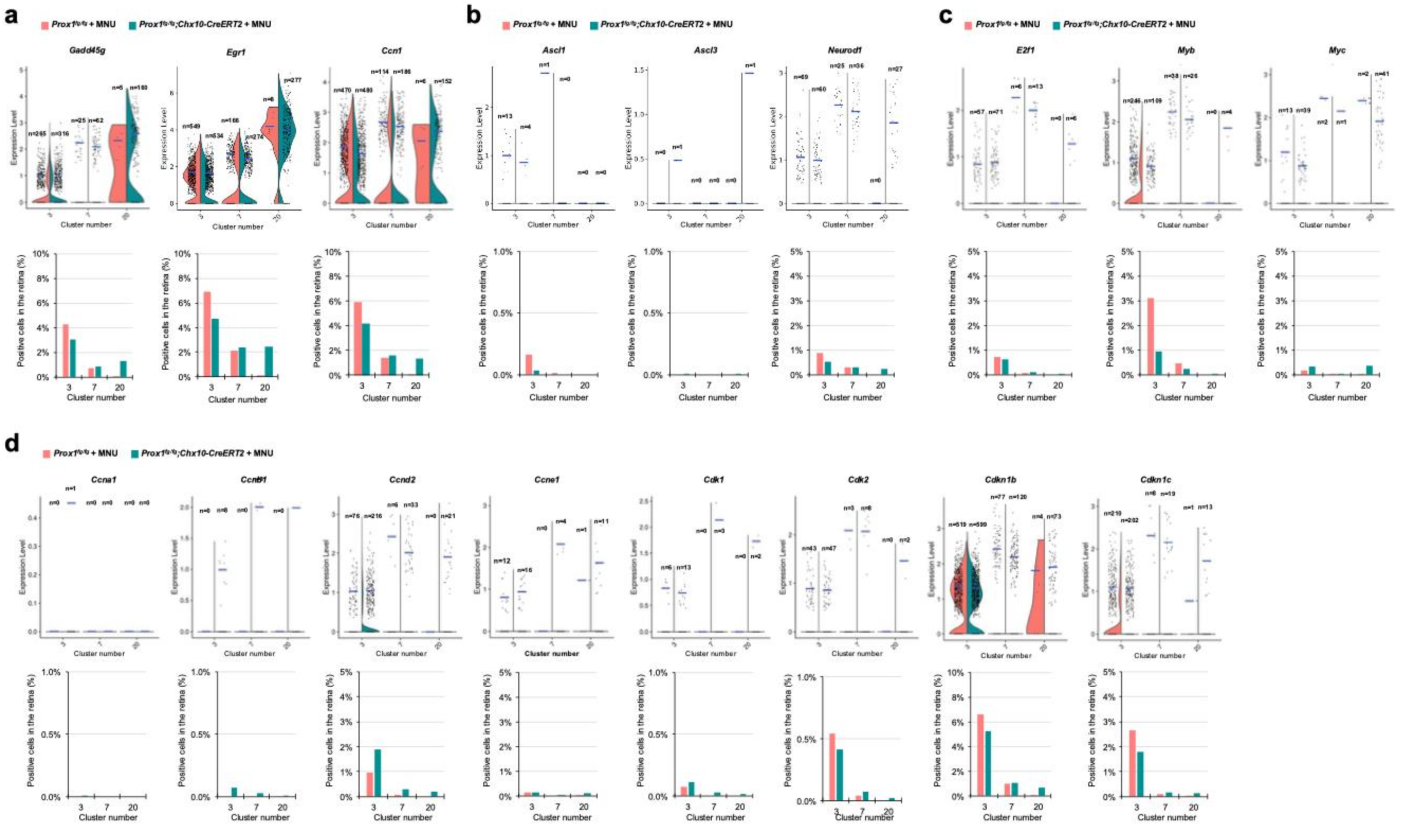

Supplementary Figure 13. Expression of cell cycle regulatory genes in cluster #20. (a) Violin plots of the cells expressing mRNA of the RPC marker, *Gadd45g*; the target of Hbegf signaling, *Egr1*; and the target of Yap/Taz transcription activators, *Ccn1*, in MG (#3 and #7) and MGPC (#20) populations. **(b)** Violin plots of the cells expressing proneural transcription factor *Ascl1*, *Ascl3*, and *Neurod1* mRNA. **(c)** Violin plots of the cells expressing *E2f1*, *Myb*, and *Myc* mRNA. **(d)** Violin plots of the cells expressing Cyclin (*Ccna1*, *Ccnb1*, *Ccnd2*, and *Ccne1*), Cdk (*Cdk1* and *Cdk2*), and Cdk inhibitor (*Cdkn1b* and *Cdkn1c*) mRNA. Each dot represents the expression level of the corresponding gene in a cell. Blue horizontal bars indicate the mean expression. Graphs in the bottom of the plots show the percentage of the mRNA-expressing cells in total retinal cells. Numbers in the violin plots represent the cells expressing the corresponding genes.

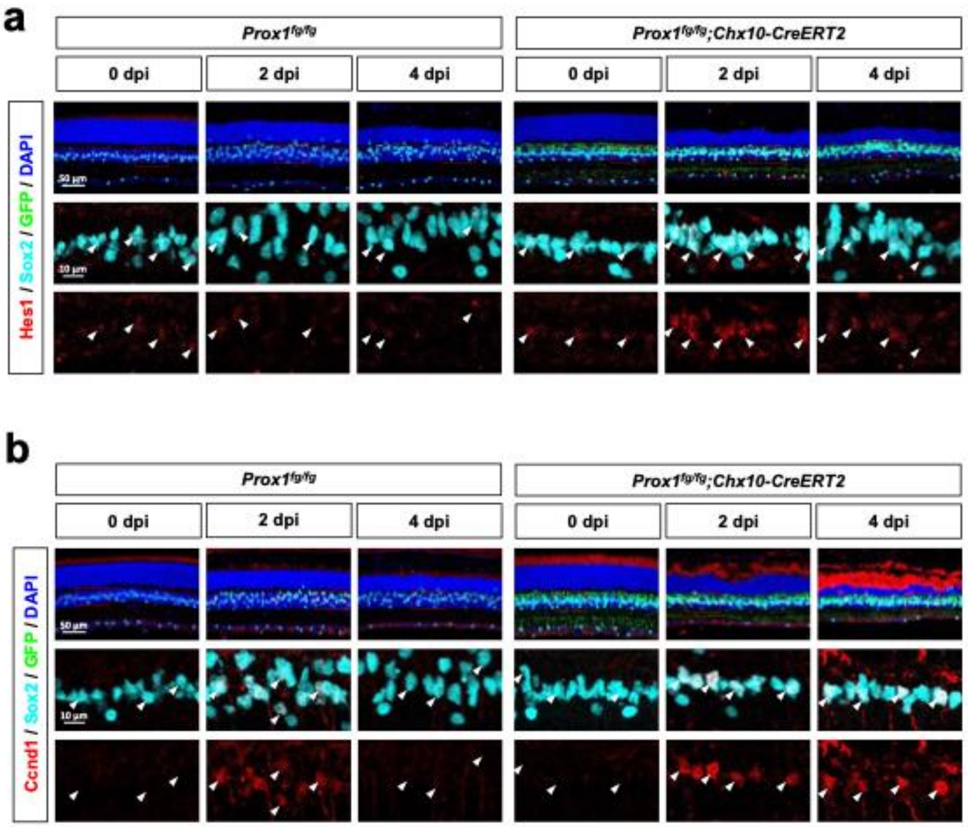

Supplementary Figure 14. Injury-induced expression of Hes1 and Ccnd1 in MGPCs of *Prox1$^{fg/fg}$;Chx10-CreERT2* mouse retinas. (a) Expression of Hes1 in Sox2-positive MG or MGPCs in the injured *Prox1$^{fg/fg}$* and *Prox1$^{fg/fg}$;Chx10-CreERT2* mouse retinas mouse retinas was investigated by immunostaining. **(b)** Expression of Ccnd1 in Sox2-positive MG or MGPCs in the injured *Prox1$^{fg/fg}$* and *Prox1$^{fg/fg}$;Chx10-CreERT2* mouse retinas mouse retinas was investigated by immunostaining. Arrowheads indicate Sox2-positive MG or MGPC nuclei.

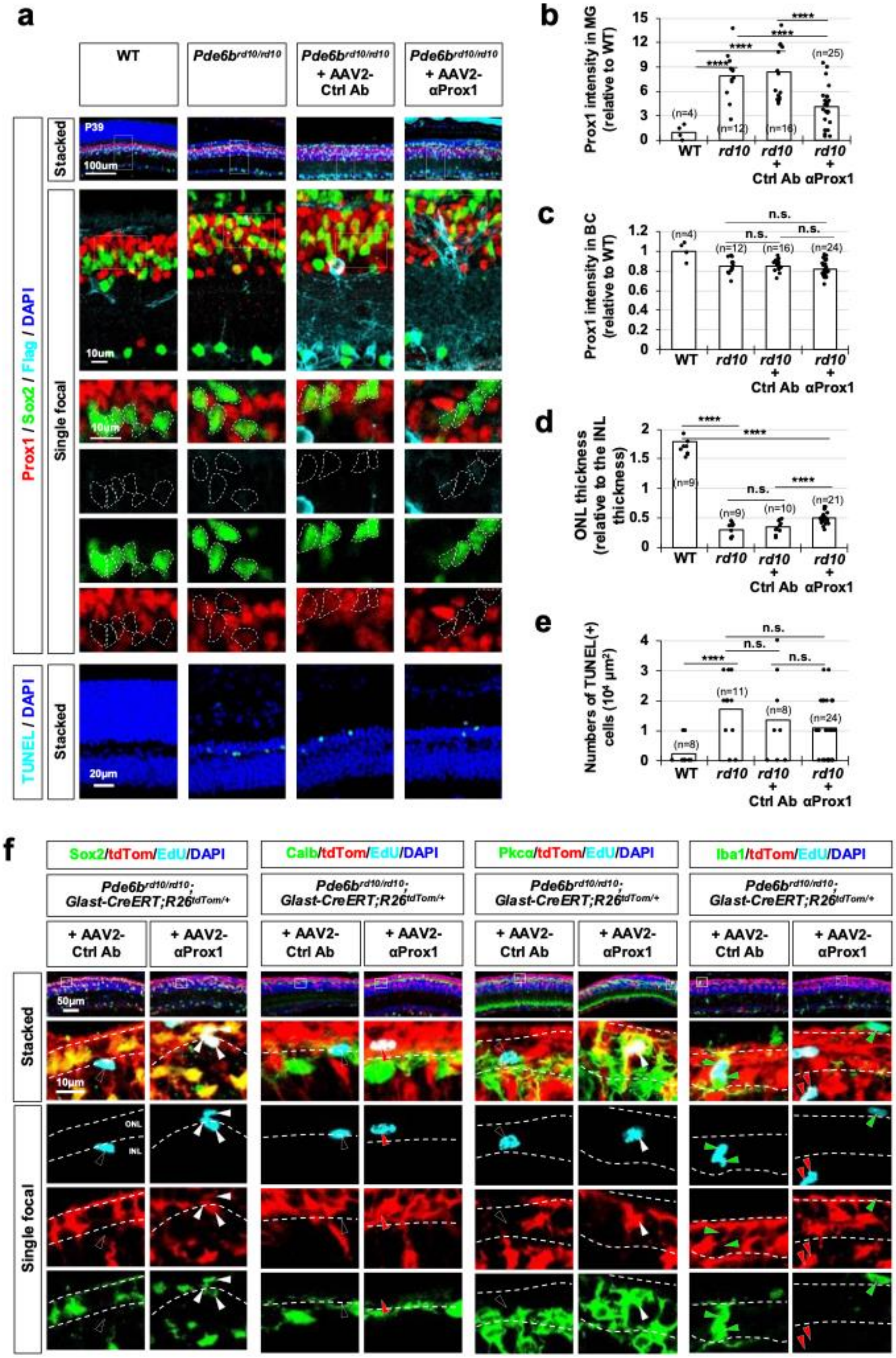
a
WT
Pde6b rd10/rd10
Pde6b rd10/rd10 + AAV2-Ctrl Ab
Pde6b rd10/rd10 + AAV2-αProx1
Prox1 / Sox2 / Flag / DAPI
Stacked
Single focal
P39
100um
10um
10μm
TUNEL / DAPI
20μm
b
Prox1 intensity in MG (relative to WT)
(n=4)
(n=12)
(n=16)
(n=25)
WT
rd10
rd10 + Ctrl Ab
rd10 + αProx1
c
Prox1 intensity in BC (relative to WT)
n.s.
(n=4)
(n=12)
(n=16)
(n=24)
d
ONL thickness (relative to the INL thickness)
(n=9)
(n=10)
(n=21)
e
Numbers of TUNEL(+) cells (10⁴ μm²)
(n=8)
(n=11)
(n=8)
(n=24)
f
Sox2/tdTom/EdU/DAPI
Calb/tdTom/EdU/DAPI
Pkcα/tdTom/EdU/DAPI
Iba1/tdTom/EdU/DAPI
Pde6b rd10/rd10; Glast-CreERT;R26 tdTom/+
+ AAV2-Ctrl Ab
+ AAV2-αProx1
50μm
10μm
ONL
INL

Supplementary Figure 15. Regeneration of retinal neurons from MG in *Pde6b$^{rd10/rd10}$* mice expressing αProx1. (a) *Pde6b$^{rd10/rd10}$* mice were intravitreally injected with AAV2 encoding FLAG-tagged control antibody (Ctrl Ab) or αProx1, as shown in Fig. 7f. The expression of the antibodies in the mouse retinas was identified by immunostaining with mouse anti-FLAG antibody. Prox1 expression in Sox2-positive MG or MGPCs (outlined by dotted lines) were also examined by co-immunostaining with rabbit anti-Prox1 and goat anti-Sox2 antibodies. The apoptotic cells in the sections were identified by TUNEL staining. **(b and c)** Prox1 intensity in MG/MGPCs (b) or BCs (c) relative to those of *Pde6b$^{rd10/rd10}$* WT littermate mice are presented in the graphs. **(d)** The thicknesses of the ONL and INL of the retinal sections were measured and relative thickness of the ONL compared to the INL of the same retinal sections is presented in the graph. **(e)** The number of TUNEL(+) apoptotic cells per retinal area is presented in the graph. Numbers of samples analyzed are shown in the graphs (data from 3 independent litters). P-values were calculated using one-sided Student's t-test (****, $p < 0.001$; n.s., not significant). **(f)** Identities of EdU-labeled cells in the AAV-injected *Pde6b$^{rd10/rd10}$* mouse retinas were investigated by immunostaining for Sox2 (MG), Calb (HC), Pkcα (BC), and Iba1 (Microglia). MG origin of the cells was determined by the expression of tdTom Cre reporter. White arrowheads, EdU-positive cells co-expressing tdTom and the markers; red arrowheads, EdU-labeled cells containing tdTom but lacking of the markers; green arrowheads, EdU-labeled cells containing the markers but lacking of tdTom; black arrowheads, EdU-labeled cells lacking of tdTom and EdU. Numbers of EdU-positive cells expressing tdTom and corresponding markers are shown in Fig. 7h.

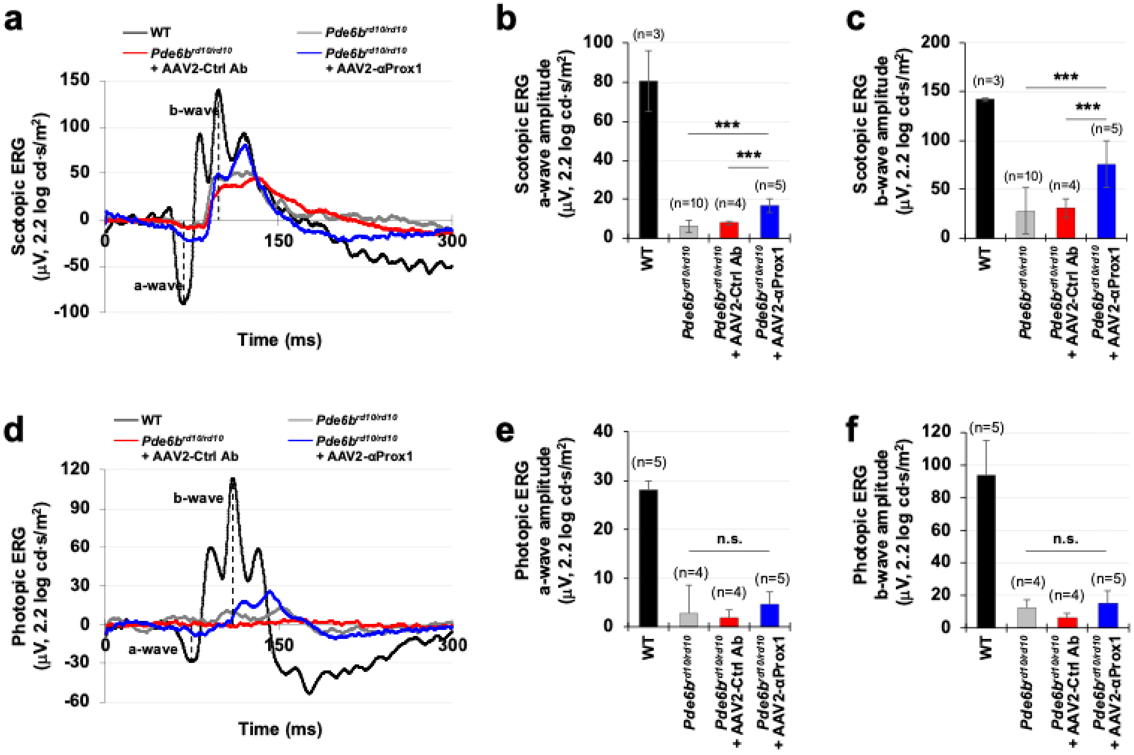

Supplementary Figure 16. Recovery of photoreceptor activity in *Pde6b*$^{rd10/rd10}$ mice expressing αProx1. (a and **d)** Scotopic and photopic ERG histograms of *Pde6b*$^{rd10/rd10}$ mice infected with the indicated AAV2 were measured at P35 (see details in Methods). Amplitudes of ERG a-waves **(b** and **e)** and b-waves **(c** and **f)** in the mouse retina. Error bars in the graphs denote SEM. Numbers of samples analyzed are shown in the graphs (data from 4 independent litters). P-values were calculated using one-sided Student's t-test (****, $p < 0.001$; n.s., not significant).

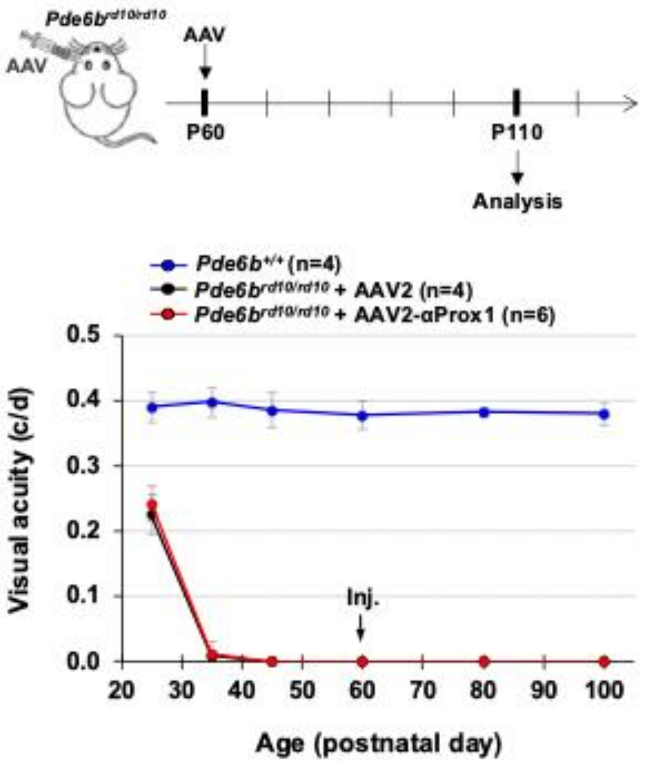

Supplementary Figure 17. AAV2-αProx1 injection was not affected to late-stage RP model mice. *Pde6b*$^{rd10/rd10}$ mice received intravitreal injections (Inj.) of AAV2 or AAV2-αProx1 ($5X10^9$ vg/eye) at P60, when they have already lost their vision completely. Visual acuities of the injected mice were measured by Optomotry.

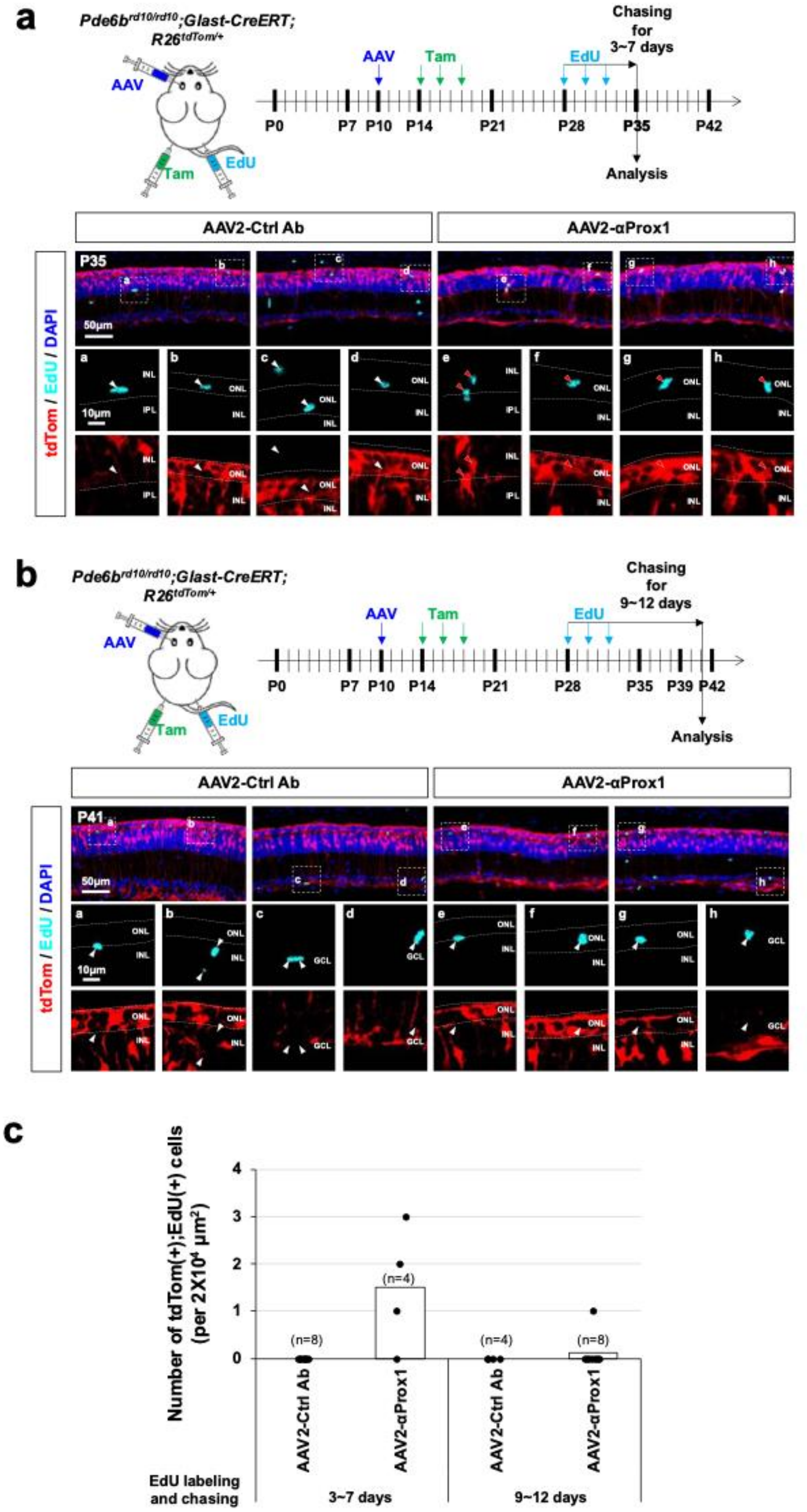
a
Pde6b^rd10/rd10;Glast-CreERT; R26^tdTom/+
AAV
Tam
EdU
Chasing for 3~7 days
P0 P7 P10 P14 P21 P28 P35 P42
Analysis
AAV2-Ctrl Ab
AAV2-αProx1
P35
50µm
10µm
tdTom / EdU / DAPI
INL
IPL
ONL
b
Pde6b^rd10/rd10;Glast-CreERT; R26^tdTom/+
Chasing for 9~12 days
P0 P7 P10 P14 P21 P28 P35 P39 P42
Analysis
P41
GCL
c
Number of tdTom(+);EdU(+) cells (per 2X10^4 µm^2)
0
1
2
3
4
(n=8)
(n=4)
(n=4)
(n=8)
AAV2-Ctrl Ab
AAV2-αProx1
EdU labeling and chasing
3~7 days
9~12 days

Supplementary Figure 18. Lifespan of newborn photoreceptors in *Pde6b$^{rd10/rd10}$* mouse retinas. (a and **b)** *Pde6b$^{rd10/rd10}$;Glast-CreERT;R26$^{tdTom/+}$* mice were intravitreally injected with AAV2 encoding FLAG-tagged Ctrl Ab or αProx1. The mice were repeatedly injected with Tam to activate CreERT in MG and EdU to labeled newborn cells in the retina as it is indicated. The mouse eyes were then isolated for the immunostaining of EdU-labeled cells in the retinal sections. MG origin of EdU-labeled cells was determined by the expression of tdTom Cre reporter. **(c)** Numbers of EdU-labeled cells remained by 3-7 days (a) or 9-12 days (b) in *Pde6b$^{rd10/rd10}$* mouse retinas are presented in the graph (data from 3 independent litters).

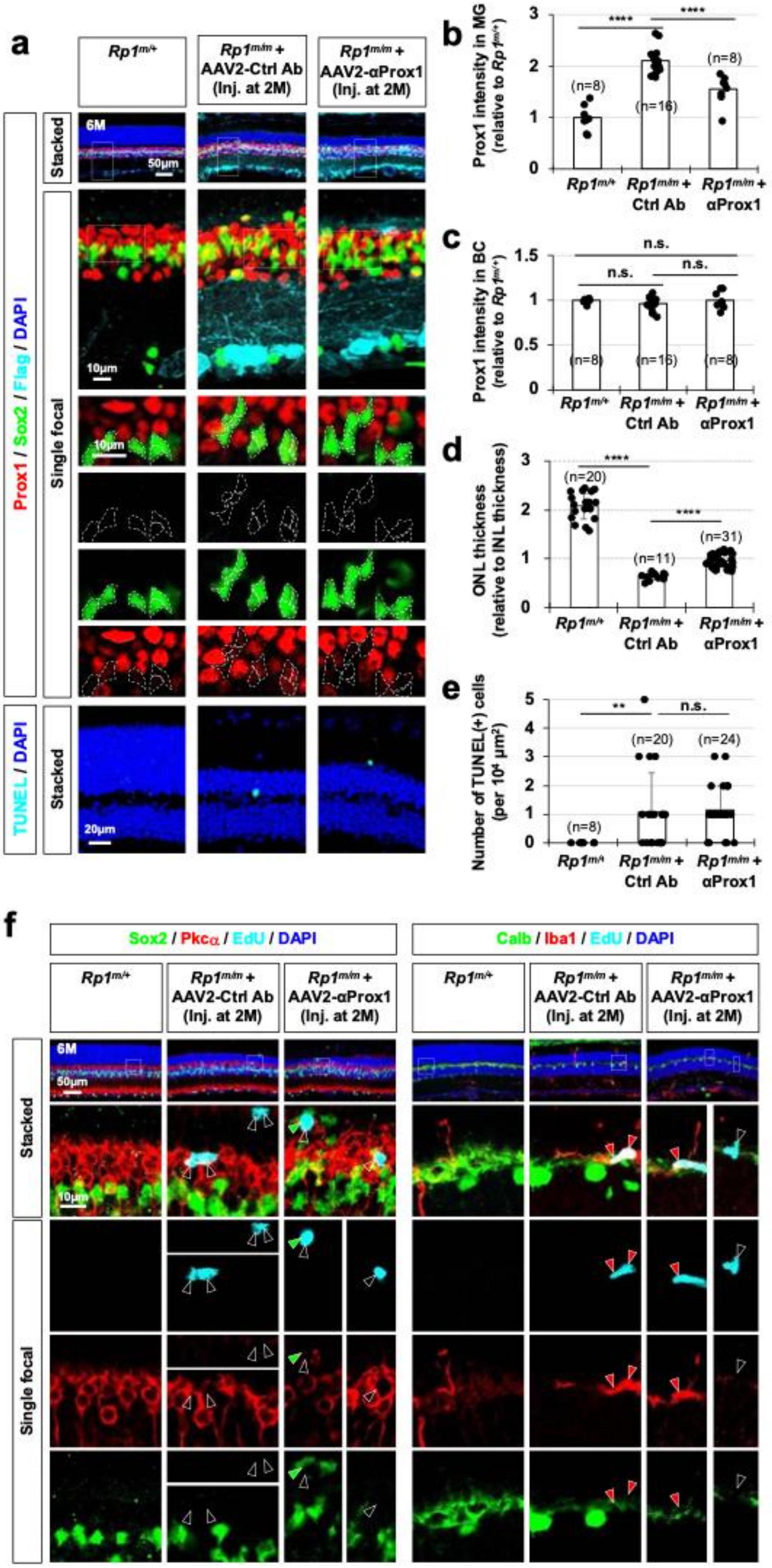
a
Rp1m/+
Rp1m/m + AAV2-Ctrl Ab (Inj. at 2M)
Rp1m/m + AAV2-αProx1 (Inj. at 2M)
Prox1 / Sox2 / Flag / DAPI
Stacked
Single focal
TUNEL / DAPI
6M
50μm
10μm
20μm
b
Prox1 intensity in MG (relative to Rp1m/+)
(n=8)
(n=16)
****
Rp1m/+
Rp1m/m + Ctrl Ab
Rp1m/m + αProx1
c
Prox1 intensity in BC (relative to Rp1m/+)
n.s.
d
ONL thickness (relative to INL thickness)
(n=20)
(n=11)
(n=31)
e
Number of TUNEL(+) cells (per 10⁴ μm²)
**
(n=24)
f
Sox2 / Pkcα / EdU / DAPI
Calb / Iba1 / EdU / DAPI

Supplementary Figure 19. Regeneration of retinal neurons in *Rp1*$^{m/m}$ mice expressing αProx1. (a) *Rp1*$^{m/m}$ mice were intravitreally injected with AAV2 encoding FLAG-tagged Ctrl Ab or αProx1 at 2M. The expression of the antibodies in the mouse retinas was then identified at 6M by immunostaining with mouse anti-FLAG antibody. Prox1 expression in Sox2-positive MG or MGPC (surrounded by dotted circles) were examined by co-immunostaining with rabbit anti-Prox1 and goat anti-Sox2 antibodies. The apoptotic cells in the sections were identified by TUNEL staining. **(b and c)** Prox1 intensity in MG/MGPCs (b) or BCs (c) relative to those of *Rp1*$^{m/+}$ littermate mice are presented in the graphs (data from 2 independent litters). **(d)** The thicknesses of the ONL and INL of the retinal sections were measured and relative thickness of the ONL compared to the INL of the same retinal sections is presented in the graph. **(e)** The number of TUNEL(+) apoptotic cells per retinal area is presented in the graph. **(f)** Identities of EdU-labeled cells in the AAV-injected *Rp1*$^{m/m}$ mouse retinas were investigated by immunostaining for Sox2 (MG or MGPC), Pkcα (BC), Calb (HC), and Iba1 (microglia). Red and green arrowheads, EdU-labeled cells containing the markers in corresponding colors; black arrowheads, EdU-labeled cells lacking of the markers. Numbers of EdU-positive cells expressing corresponding markers are shown in Fig. 8f. Numbers of samples analyzed are shown in the graphs (data from 2 independent litters). P-values were calculated using one-sided Student's t-test (****, $p<0.001$; **, $p<0.01$; n.s., not significant).

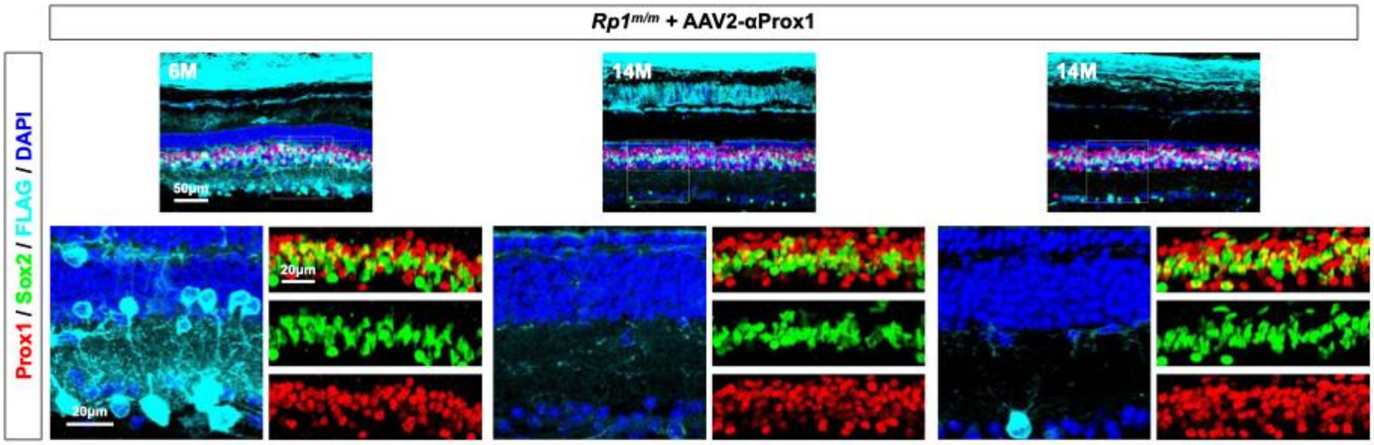

Supplementary Figure 20. Loss of αProx1 expression in AAV2-αProx1-infected *Rp1$^{m/m}$* mice. *Rp1$^{m/m}$* mice (2 months-old) were intravitreally injected with AAV2-αProx1 and the expression of αProx1 in the mouse retinas was assessed at 6 months (6M) or 14 months (14M) age of the mice by immunostaining for the FLAG tag of the antibody. Sox2-positive MG and Prox1-expressing cells in the mouse retinas were also identified by co-immunostaining of the retinal sections.

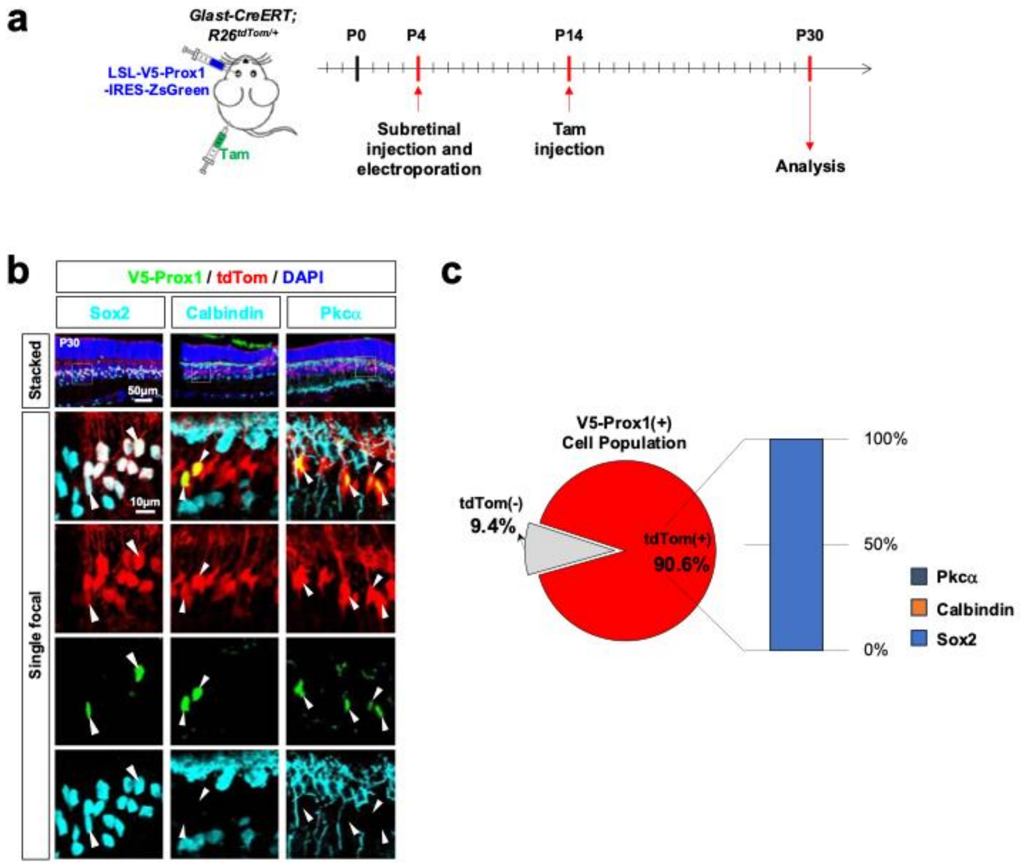

Supplementary Figure 21. Prox1 overexpression in MG does not induce fate conversion to retinal neurons. (a) Plasmid DNA (2.5 µg, 0.5 µL) encoding LSL-V5-Prox1-IRES-ZsGreen was injected into the subretinal space of P4 *Glast-CreERT;R26*$^{tdTom/+}$ mice. Tam was administered to activate CreERT, which excised the LSL cassette in the *R26*^{tdTom} locus, leading to tdTom expression in MG. Simultaneously, Cre-mediated excision of the LSL sequence in the plasmid enabled the expression of V5-Prox1 and ZsGreen in transfected MG. **(b)** At P30, the identity of V5-Prox1 and ZsGreen co-expressing cells was assessed by immunostaining for cell type-specific markers: Sox2 (MG marker), Calbindin (HC and an AC subset), and Pkcα (rod BC marker). **(c)** The composition of V5-Prox1-positive cells in the mouse retina is summarized in the accompanying graph.

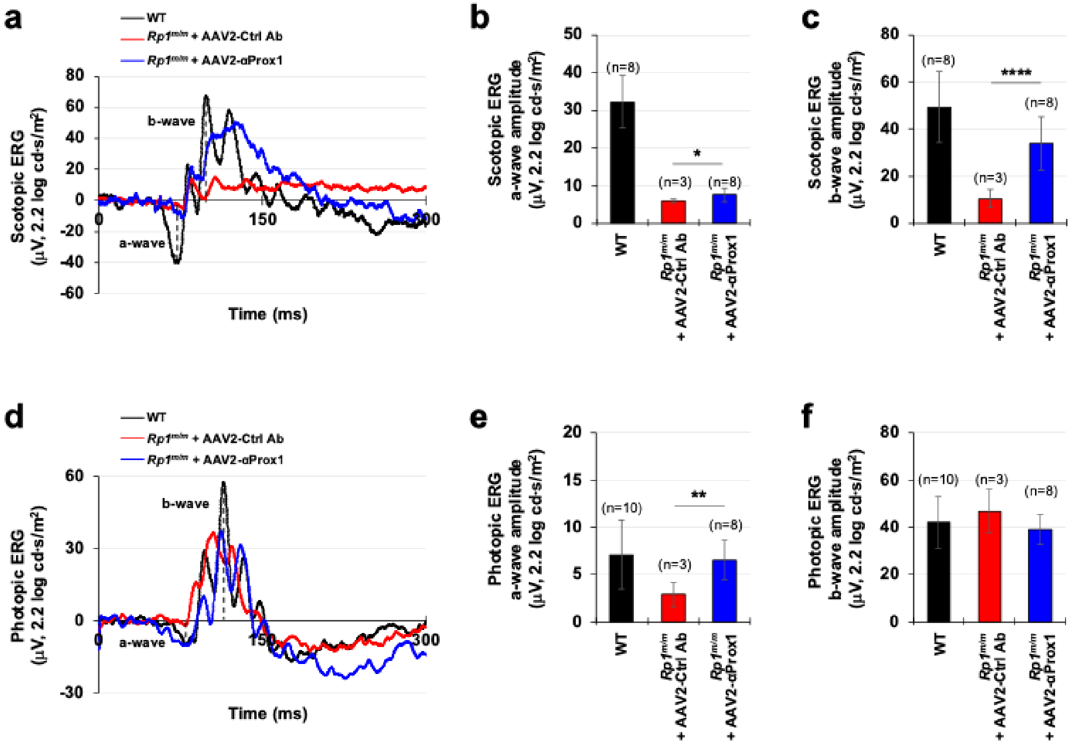

Supplementary Figure 22. Recovery of photoreceptor activity in RP model mice expressing αProx1. (a and **d)** Scotopic and photopic ERG histograms of 6 months-old *Rp1*$^{m/m}$ mice infected with the indicated AAV2 were measured. Amplitudes of ERG a-waves **(b** and **e)** and b-waves **(c** and **f)** in the mouse retina. Error bars in the graphs denote SEM. Numbers of samples analyzed are shown in the graph (data from 2 independent litters). P-values were calculated using one-sided Student's t-test (* p<0.05; ** p<0.01).

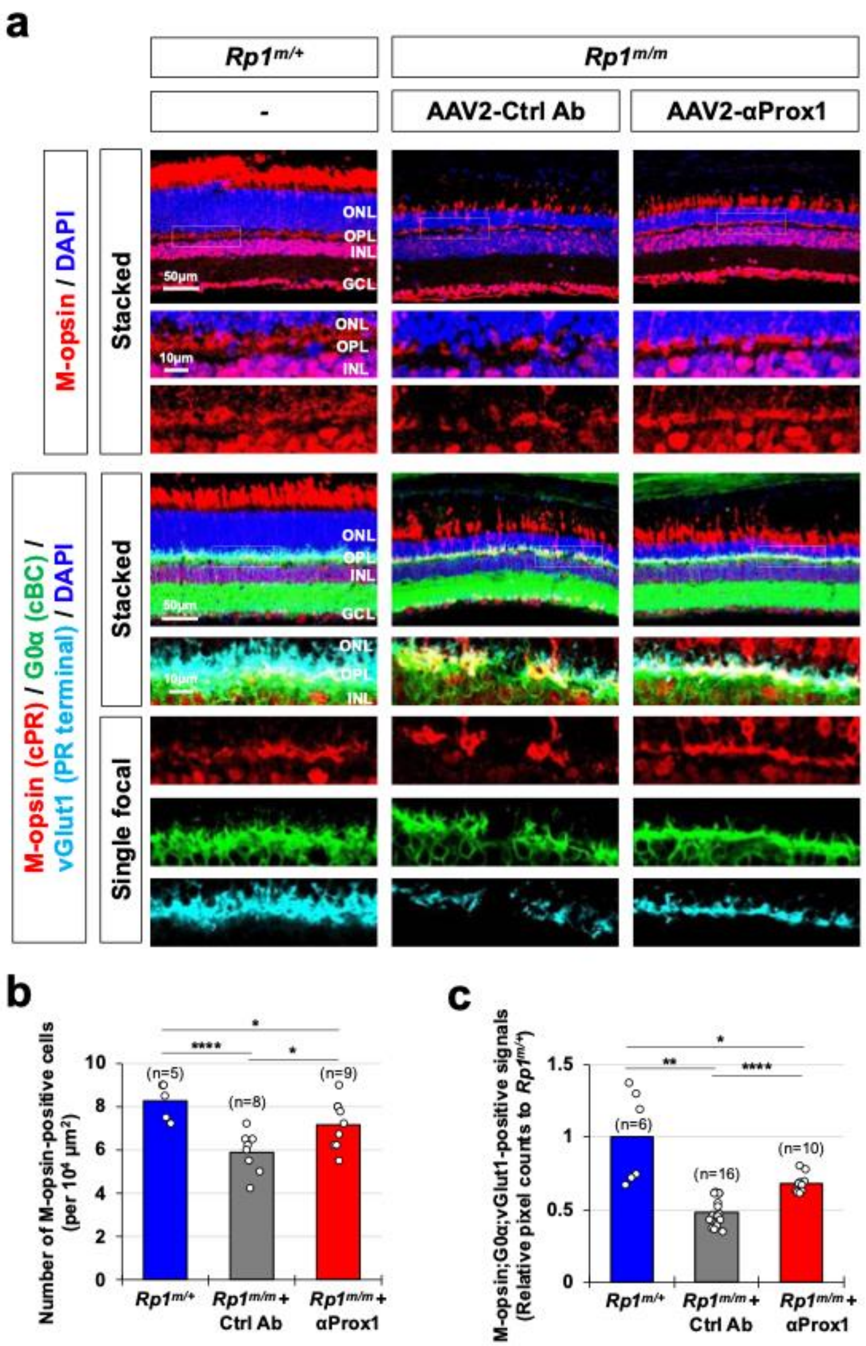

Supplementary Figure 23. Preservation of cone photoreceptors in *Rp1*$^{m/m}$ mice expressing αProx1. (a) *Rp1*$^{m/m}$ mice were intravitreally injected with AAV2 encoding FLAG-tagged Ctrl Ab or αProx1, as shown in Fig. 8d Distribution of cPRs in the mouse retinas were examined by immunostaining of M-opsin. Synaptic terminals of the PRs and their post-synaptic targets, i.e., cone bipolar cells (cBCs), are visualized by co-immunostaining of respective markers, vGlut1 and G0α. **(b)** Number of M-opsin-positive cPRs were counted and presented in the graph. **(c)** The pixel counts of M-opsin;G0α;vGlut1 co-stained signals were measured from the immunostaining images and depict in the graph. Numbers of samples analyzed are shown in the graph (data from 2 independent litters). P-values were calculated using one-sided Student's t-test (* $p<0.05$; ** $p<0.01$; **** $p<0.001$).

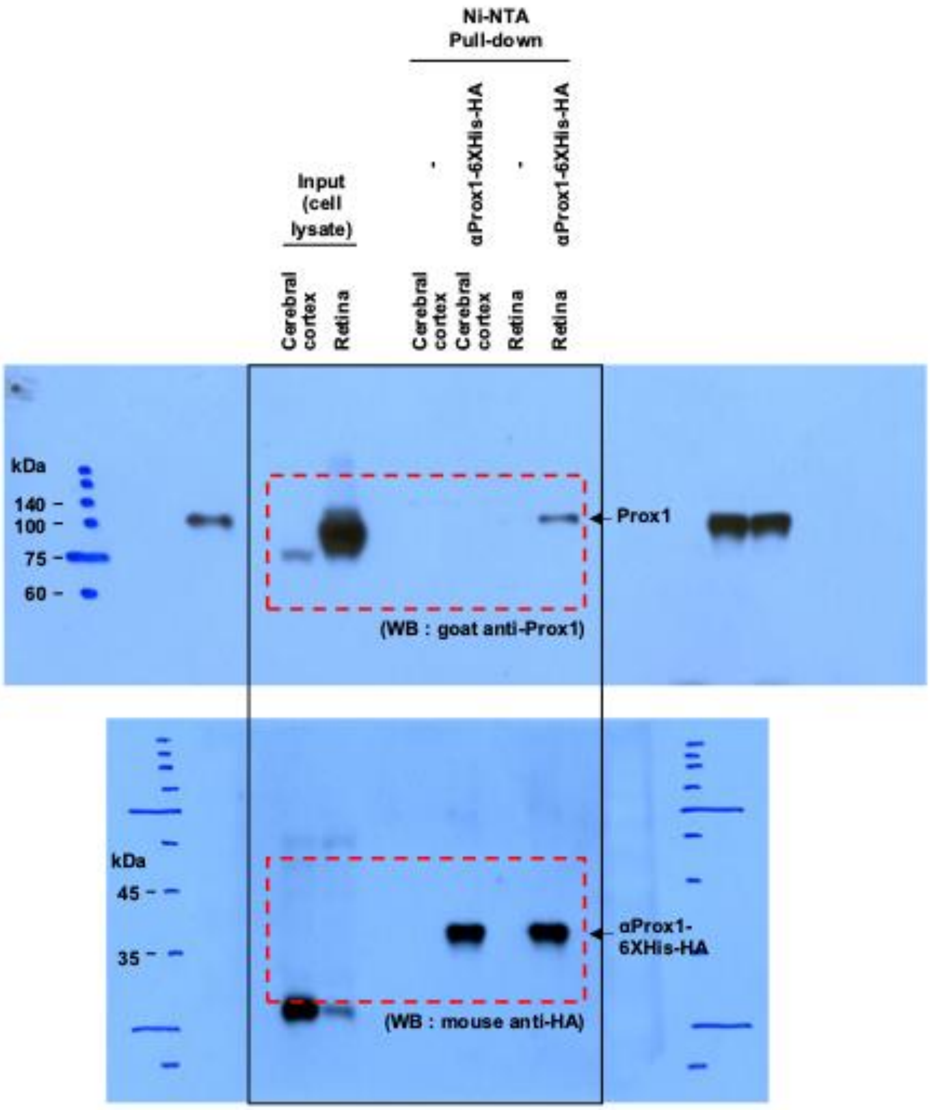

Supplementary Figure 24. Uncropped images of the blots used in Supplementary Figure 9A.

Supplementary Table 1. List of materials and resources used in this study.

REAGENT or RESOURCE	SOURCE	IDENTIFIER
Antibodies [dilution for application]		
Rabbit polyclonal anti-Prox1 (applied to IHC [1:1,000] and IVT)	Merck Milipore	ABN278
Mouse monoclonal anti-Prox1 (applied to IHC [1:500])	abcam	ab92825
Goat polyclonal anti-Prox1 (applied to WB [1:50])	R&D system	AF2727
Chicken polyclonal anti-GFP (IHC [1:2,000], WB [1:10,000])	abcam	ab13970
Goat polyclonal anti-Sox2 (IHC [1:100])	R&D system	AF2018
Rabbit polyclonal anti-Sox2 (IHC [1:200])	abcam	ab97959
Rabbit polyclonal anti-Sox9 (IHC [1:200])	abcam	ab185230
Mouse monoclonal anti-p27[Kip1] (IHC [1:100])	BD	610242
Rabbit polyclonal anti-Glutamate sythetase (IHC [1:200])	Sigma	G2781
Mouse monoclonal anti-Glutamate sythetase (GS-6) (IHC [1:200])	Merck Milipore	MAB302
Rabbit polyclonal anti-Recoverin (IHC [1:200])	Chemicon	AB5585
Mouse monoclonal anti-Rhodopsin (1D4) (IHC [1:200])	Millipore	MAB5356
Rabbit polyclonal anti-opsin (Red/Green) (IHC [1:200])	Merck Millipore	AB5405
Goat polyclonal anti-Otx2 (IHC [1:200])	R&D system	AF1979-SP
Mouse monoclonal anti-Calbindin (D-28K) (IHC [1:200])	Sigma	C9848
Rabbit polyclonal anti-Calbindin (D-28K) (IHC [1:200])	swant	CB38a
Mouse monoclonal anti-Chx10 (E-12) (IHC [1:200])	Santa Cruz	sc-365519
Rabbit polyclonal anti-PKCα (C-20) (IHC [1:200])	Santa Cruz	sc-208
Mouse monoclonal anti-Goalpha (IHC [1:200])	Merck Milipore	MAB3073
Rabbit polyclonal anti-PAX6 (IHC [1:200])	Biolegend	901301
Mouse monoclonal anti-HuC/HuD (16A11) (IHC [1:200])	Thermo Scientific	A-21271
Rabbit polyclonal anti-Brn3a (IHC [1:100])	SYSY	411003
Mouse monoclonal anti-ASCL1 (D-7) (IHC [1:50])	Santa Cruz	sc-374104
Rabbit polyclonal anti-Iba1 (IHC [1:500])	Wako	019-19741
Guineapig polyclonal anti-Iba1 (IHC [1:500])	SYSY	234 004
Rabbit polyclonal anti-GFAP (IHC [1:200])	abcam	ab48050
Mouse monoclonal anti-Cyclin D1 (HD11) (IHC [1:100])	Santa Cruz	sc-246
Rabbit monoclonal anti-Hes1 (D6P2U) (IHC [1:20])	Cell signaling	11988
Guinea pig polyclonal anti-vGlut1 (IHC [1:200])	Sigma	AB5905
Chicken polyclonal anti-β-galactosidase (IHC [1:100])	abcam	ab9361
Mouse monoclonal anti-FLAG (M2) (IHC [1:50])	Sigma	F1804
Mouse monoclonal anti-HA (16B12) (WB [1:1,000])	Biolegend	901513
Normal Rabbit IgG	Cell signaling	2729
anti-PROX1 scFv-6XHis-HA (1A11)	This paper	N/A
Alexa Fluor 488 Donkey Anti-Mouse IgG (H+L)	Jackson ImmunoResearch	715-546-150
Alexa Fluor 488 Donkey Anti-Rabbit IgG (H+L)	Jackson ImmunoResearch	711-546-152

Alexa Fluor 488 Donkey Anti-Goat IgG (H+L)	Jackson ImmunoResearch	705-546-147
Alexa Fluor 488 Donkey Donkey Anti-Chicken IgY (IgG) (H+L)	Jackson ImmunoResearch	703-546-155
Cyanine Cy™3 Donkey Anti-Mouse IgG (H+L)	Jackson ImmunoResearch	715-166-150
Cyanine Cy™3 Donkey Anti-Rabbit IgG (H+L)	Jackson ImmunoResearch	711-166-152
Cyanine Cy™3 Donkey Anti-Goat IgG (H+L)	Jackson ImmunoResearch	705-166-147
Alexa Fluor 647 Donkey Anti-Mouse IgG (H+L)	Jackson ImmunoResearch	715-606-150
Alexa Fluor 647 Donkey Anti-Rabbit IgG (H+L)	Jackson ImmunoResearch	711-606-152
Alexa Fluor 647 Donkey Anti-Goat IgG (H+L)	Jackson ImmunoResearch	705-606-147
Normal Donkey Serum	Jackson ImmunoResearch	017-000-121
Bacterial and virus strains		
DH5α	Enzynomics	CP011
AAV2-αProx1 (AAV2/2-EF1α-IL2'SP-anti-PROX1-scFv(1A11)-FLAG)	This paper	N/A
AAV2-Ctrl Ab (AAV2/2-EF1α-IL2'SP-Control scFv(N1T1D6)-FLAG)	This paper	N/A
Chemicals, peptides, and recombinant proteins		
Tamoxifen	Sigma	T5648
MNU (N-Nitroso-N-methylurea)	Spectrum chemical	N2939
NMDA (N-Methyl-D-aspartic acid)	Sigma	M3262
2,2,2-Tribromoethanol	Sigma	T48402
Hoechst33342	invitrogen	H1399
In situ Cell Death Detection Kit, Fluorescein	Roche	11684795910
In situ Cell death Detection Kit, TMR-red	Roche	12156792910
EdU (5-ethynyl-2'-deoxyuridine)	invitrogen	E10187
Click-iT EdU Alexa Fluor 647 Imaging Kit	invitrogen	C10340
3XFLAG peptide	Sigma	F4799
FLAG-Prox1 protein	This paper	N/A
Protein G Sepharose 4 Fast Flow	GE Healthcare	17-0618-01
Ni-NTA Chelating Agarose CL-6B	Peptron	1103
O.C.T. Compound	Scigen	4586
Fluorescence Mounting Medium	Dako	S3023
RNAscope® Intro Pack for Multiplex Fluorescent Reagent Kit v2- Mm with TSA Vivid Dyes	ACDbio	323280
RNA-Protein Co-Detection Ancillary Kit	ACDbio	323180
AllPrep DNA/RNA/Protein Mini kit	Qiagen	80004
DMEM/High glucose	Hyclone	SH30243
Fetal Bovine Serum	GIBCO	12484010
Penicillin-Streptomycin	Invitrogen	15140
GenJet In Vitro DNA Transfection Reagent (Ver. II)	SignaGen	SL100489
Grace's insect medium	GIBCO	11605-094
Poloxamer 188 solution	Sigma	P5556
Gentamicin	GIBCO	15710-064
Cellfectin II Reagent	GIBCO	10362
Experimental models: Cell lines		

Human: HeLa cells	ATCC	RRID: CVCL _0030
Insect: Sf9 cells	ATCC	RRID: CVCL _0549
Experimental models: Organisms/strains		
Mouse: Prox1$^{tm1.1Fuma}$ (Prox1fg)	RIKEN CDB	CDB0482K
Mouse: Dkk3^{tm1Tfur} (Dkk3-Cre)	RIKEN BRC	RBRC05427
Mouse: Tg(Chx10-cre/ERT2)G7Tfur (Chx10-CreERT2)	RIKEN BRC	RBRC05565
Mouse: Tg(Slc1a3-cre/ERT)1Nat/J (Glast-CreERT)	Jackson Laboratory	012586
Mouse: Tg(Crx-cre/ERT2)1Tfur (Crx-CreERT2)	RIKEN BRC	RBRC05559
Mouse: B6.CXB1-Pde6b^{rd10}/J (Pde6b^{rd10})	Jackson Laboratory	004297
Mouse: C57BL/6J-Rp1^{tvrm64}/PjnMmjax (Rp1^{tvrm})	Jackson Laboratory	043579
Mouse: B6.129P2-Gt(ROSA)26Sor$^{tm1(DTA)Lky}$/J (R26^{DTA})	Jackson Laboratory	009669
Mouse: B6.Cg-Gt(ROSA)26Sor$^{tm14(CAG-tdTomato)Hze}$/J (R26t dTom)	Jackson Laboratory	007914
Mouse: B6;129S-Gt(ROSA)26Sor/J (R26^{LacZ})	Jackson Laboratory	002073
Zebrafish: Tg(gfap:EGFP)		ZDB-TGCONSTRCT-070410-2
Oligonucleotides		
RT-qPCR: Prox1 Forward: CACTCATAAAGTCTGAGTGT	This paper	N/A
RT-qPCR: Prox1 Reverse: GGTGTAAAAGAACATGAGTT	This paper	N/A
RT-qPCR: GS Forward: TTATGGGAACAGACGGCCAC	This paper	N/A
RT-qPCR: GS Reverse: GGTAGTGAGCCTCCACGATG	This paper	N/A
RT-qPCR: Pkca Forward: AAGGCAACATGGAACTCAGG	This paper	N/A
RT-qPCR: Pkca Reverse: TGTCAGCAAGCATCACCTTC	This paper	N/A
RT-qPCR: Rhodopsin Forward: GCAGCCTTGGTCTCTGTCTAC	This paper	N/A
RT-qPCR: Rhodopsin Reverse: GAAGTTGCTCATCGGCTTGC	This paper	N/A
RT-qPCR: Actb1 Forward: TTTCCAGCCTTCCTTCTTGG	This paper	N/A
RT-qPCR: Actb1 Reverse: GGCATAGAGGTCTTTACGGATG	This paper	N/A
RNAscope probe Probe-Mm-Prox1-C1	ACDbio	488591
Recombinant DNA		
pCAG-V5-PROX1-IRES-EGFP	Lee et al., 2019	N/A

pCAG-V5-OTX2-IRES-EGFP	Lee et al., 2019	N/A
pCAG-LoxP-STOP-LoxP-V5-PROX1-IRES-ZsGreen	This paper	N/A
Software and algorithms		
Fluoview 4.0	Olympus Corporation	N/A
Multi Gauge V3.0	Fujifilm Life Science	N/A
OptoMotry HD System	CerebralMechanics	http://cerebralmechanics.com/
Phoenix MICRON Ganzfeld ERG	Phoenix-MICRON	https://phoenixmicron.com/
Python 3.11	N/A	https://www.python.org/downloads/release/python-3110/
Monocle3	Cole Trapnell	
Seurat	SatijaLab	
StarDist	N/A	https://github.com/stardist/stardist

Supplementary Material 1. Electronic medical record (EMR) of healthy donor.

[redacted]

Discharge Summary [redacted] **at 10/10/2022 10:47 AM**

[redacted]

Service: —
Date of Service: 10/10/2022 10:47 AM

Author Type: Physician
Note Type: Discharge Summary

Status: Signed

[redacted]

DISCHARGE SUMMARY
Date of service: 10/10/2022

[redacted]
83 y.o.
[redacted]

Admit date: 10/4/2022

Discharge date: 10/10/2022

Admission Diagnoses: neutropenic fever

Discharge Diagnoses:
Principal Problem:
- Neutropenic fever (HRC)

Active Problems:
- Hyperlipidemia (HRC)
- Idiopathic peripheral neuropathy
- Mild intermittent asthma without complication (HRC)
- Chronic diastolic heart failure (HRC)
- Coronary artery disease involving native coronary artery of native heart without angina pectoris (HRC)
- Essential tremor (HRC)
- MDS (myelodysplastic syndrome) (HRC)
- Pancytopenia (HRC)

Hospital Course:
83 y.o. female with pmhx HLD, Asthma, CHF, MDS wtih blasts nearing threshold of transformation to AML undergoing chemo last dose 10 days, started hydrea in anticipation of venetoclax admitted for neutropenic fever with CAP.
Has since transitioned to hospice

Active problems:
Patient Active Hospital Problem List:

Neutropenic fever (HRC) (10/5/2022)
MDS (myelodysplastic syndrome) (HRC) (8/16/2022)

[redacted]

Supplementary Material 2. Electronic medical record (EMR) of RP donor.

[redacted]

[redacted] [redacted] Date of Service: 9/28/2022 12:14 AM
Signed

Signed

[redacted] **79 y o** **female** [redacted]

CHIEF COMPLAINT:
Cardiac Arrest

HPI
History is limited given the condition of the patient

History provided by husband.

79-year-old female presents with unresponsiveness. Husband states that the patient had a fairly normal day today and then tarted vomiting tonight On their way in he ha become unre pon ive
ALLERGIES:
Lisinopril and Sulfa (sulfonamide antibiotics)

CURRENT MEDICATIONS acetaminophen (TYLENOL E TRA STRGTH) 500 mg tablet
amLODIPine (NORVASC) 5 mg tablet
aspirin (ECOTRIN) 81 mg enteric coated tablet
atenoloL (TENORMIN) 50 mg tablet
chlorthalidone (HYGROTON) 25 mg tablet
cholecalciferol (VITAMIN D-3) 2,000 unit capsule
levothyroxine (Euthyrox) 100 mcg tablet
sertraline (ZOLOFT) 50 mg tablet
triamcinolone (ARISTOCORT; KENALOG) 0 1 % cream

PROBLEM LIST:
HYPOTHYROIDISM

Morbid obesity with BMI of 40.0-44.9, adult (HC)

Pulmonary artery hyperten ion (HC)

OSA (obstructive sleep apnea)

Retiniti pigmento a

PAD (peripheral artery disease) (HC)

Venou in ufficiency of left leg

Left atrial enlargement

O teopenia

Hyperlipemia

Major depre ive di order, recurrent epi ode, moderate (HC)

[redacted]
