# Supplementary material for: Restoration of retinal regenerative potential of Müller glia by disrupting intercellular Prox1 transfer
Source: Nat Commun. 2025 Mar 25;16:2928. doi: 10.1038/s41467-025-58290-8 (PMC11937340; doi:10.1038/s41467-025-58290-8)
Supplement: Supplementary file 2 — Reporting Summary [file 41467_2025_58290_MOESM2_ESM.pdf]

nature portfolio

Corresponding author(s): Jin Woo Kim

Last updated by author(s): 02/20/2025

Reporting Summary

Nature Portfolio wishes to improve the reproducibility of the work that we publish. This form provides structure for consistency and transparency in reporting. For further information on Nature Portfolio policies, see our Editorial Policies and the Editorial Policy Checklist.

Statistics

For all statistical analyses, confirm that the following items are present in the figure legend, table legend, main text, or Methods section.

n/a	Confirmed	
☐	☒	The exact sample size (*n*) for each experimental group/condition, given as a discrete number and unit of measurement
☐	☒	A statement on whether measurements were taken from distinct samples or whether the same sample was measured repeatedly
☐	☒	The statistical test(s) used AND whether they are one- or two-sided<br>*Only common tests should be described solely by name; describe more complex techniques in the Methods section.*
☐	☒	A description of all covariates tested
☐	☒	A description of any assumptions or corrections, such as tests of normality and adjustment for multiple comparisons
☐	☒	A full description of the statistical parameters including central tendency (e.g. means) or other basic estimates (e.g. regression coefficient) AND variation (e.g. standard deviation) or associated estimates of uncertainty (e.g. confidence intervals)
☐	☒	For null hypothesis testing, the test statistic (e.g. *F*, *t*, *r*) with confidence intervals, effect sizes, degrees of freedom and *P* value noted<br>*Give P values as exact values whenever suitable.*
☒	☐	For Bayesian analysis, information on the choice of priors and Markov chain Monte Carlo settings
☒	☐	For hierarchical and complex designs, identification of the appropriate level for tests and full reporting of outcomes
☒	☐	Estimates of effect sizes (e.g. Cohen's *d*, Pearson's *r*), indicating how they were calculated

Our web collection on statistics for biologists contains articles on many of the points above.

Software and code

Policy information about availability of computer code

Data collection	FV31S-SW (Olympus); DP controller (Olympus); OptoMotry HD System (CerebralMechanics); Phoenix MICRON Ganzfeld ERG (Phoenix); qTOWER³ (Analytik Jena)
Data analysis	Prism v5.0 (GraphPad); Multi Gauge v3.0 (Fujifilm); Python 3.11, Monocle3, Seurat

For manuscripts utilizing custom algorithms or software that are central to the research but not yet described in published literature, software must be made available to editors and reviewers. We strongly encourage code deposition in a community repository (e.g. GitHub). See the Nature Portfolio guidelines for submitting code & software for further information.

Data

Policy information about availability of data

All manuscripts must include a data availability statement. This statement should provide the following information, where applicable:

- Accession codes, unique identifiers, or web links for publicly available datasets
- A description of any restrictions on data availability
- For clinical datasets or third party data, please ensure that the statement adheres to our policy

All data generated or analyzed during this study are included in this published article (and its supplementary files). Source data are also provided as a Source Data file. The scRNA-seq dataset generated in this study has been deposited in Gene Expression Omnibus (GEO) under the accession numbers GSE290239 and GSE290470. We also deposited the dataset the Korea BioData Station under the under accession numbers KAP240958 and KAP240959.

Research involving human participants, their data, or biological material

Policy information about studies with human participants or human data. See also policy information about sex, gender (identity/presentation), and sexual orientation and race, ethnicity and racism.

Reporting on sex and gender	Females
Reporting on race, ethnicity, or other socially relevant groupings	Caucasian
Population characteristics	Please find the information in the enclosed Emergent Medical Data (EMD) in Supplementary Information.
Recruitment	Donors pledged to donate their eyes after death
Ethics oversight	Internal Review Board Committee of Lions Gift of Sight

Note that full information on the approval of the study protocol must also be provided in the manuscript.

Field-specific reporting

Please select the one below that is the best fit for your research. If you are not sure, read the appropriate sections before making your selection.

☒ Life sciences ☐ Behavioural & social sciences ☐ Ecological, evolutionary & environmental sciences

For a reference copy of the document with all sections, see nature.com/documents/nr-reporting-summary-flat.pdf

Life sciences study design

All studies must disclose on these points even when the disclosure is negative.

Sample size	Sample size information is provided in each figure.
Data exclusions	None
Replication	The number of independent experimental trial is described in each figure legend. Independent experiments were performed at least three times.
Randomization	For in vitro experiment, samples were treated in random order. For animal experiment, littermate mice were randomly allocated to experimental and control groups, and mice were exposed to the same environmental condition.
Blinding	Sample selection and quantitative analyses of the data were done a blinded fashion, and the samples were matched after quantification.

Reporting for specific materials, systems and methods

We require information from authors about some types of materials, experimental systems and methods used in many studies. Here, indicate whether each material, system or method listed is relevant to your study. If you are not sure if a list item applies to your research, read the appropriate section before selecting a response.

Materials & experimental systems

n/a	Involved in the study	
☐	☒	Antibodies
☐	☒	Eukaryotic cell lines
☒	☐	Palaeontology and archaeology
☐	☒	Animals and other organisms
☒	☐	Clinical data
☒	☐	Dual use research of concern
☒	☐	Plants

Methods

n/a	Involved in the study	
☒	☐	ChIP-seq
☐	☒	Flow cytometry
☒	☐	MRI-based neuroimaging

Antibodies

Antibodies used	Detailed information on antibodies used in this study is provided in Supplementary Table 1.

Validation	Detailed information on validation of antibodies used in this study is provided in Supplementary Table 1.

Eukaryotic cell lines

Policy information about cell lines and Sex and Gender in Research

Cell line source(s)	HeLa (RRID: CVCL_0030) cell lines were purchased from the American Type Culture Collection (ATCC).
Authentication	None
Mycoplasma contamination	Cell-lines used in this study was confirmed to be negative to mycoplasma contamination.
Commonly misidentified lines (See ICLAC register)	None

Animals and other research organisms

Policy information about studies involving animals; ARRIVE guidelines recommended for reporting animal research, and Sex and Gender in Research

Laboratory animals	All mouse strains used in this study are provided in Supplementary table 1.
Wild animals	None
Reporting on sex	Both male and female mice were used in this study.
Field-collected samples	None
Ethics oversight	All of the animals were handled according to approved institutional animal care and use committee (IACUC) protocols (KA13-130 and KA2019-14) of Korea Advanced Institute of Science and Technology (KAIST).<br>All zebrafish care and husbandry were performed in compliance with the guidelines of the Korea Research Institute of Bioscience and Biotechnology (KRIBB) IACUC (KRIBB-AEC-20235).

Note that full information on the approval of the study protocol must also be provided in the manuscript.

Plants

Seed stocks	*Report on the source of all seed stocks or other plant material used. If applicable, state the seed stock centre and catalogue number. If plant specimens were collected from the field, describe the collection location, date and sampling procedures.*
Novel plant genotypes	*Describe the methods by which all novel plant genotypes were produced. This includes those generated by transgenic approaches, gene editing, chemical/radiation-based mutagenesis and hybridization. For transgenic lines, describe the transformation method, the number of independent lines analyzed and the generation upon which experiments were performed. For gene-edited lines, describe the editor used, the endogenous sequence targeted for editing, the targeting guide RNA sequence (if applicable) and how the editor was applied.*
Authentication	*Describe any authentication procedures for each seed stock used or novel genotype generated. Describe any experiments used to assess the effect of a mutation and, where applicable, how potential secondary effects (e.g. second site T-DNA insertions, mosiacism, off-target gene editing) were examined.*

Flow Cytometry

Plots

Confirm that:

☒ The axis labels state the marker and fluorochrome used (e.g. CD4-FITC).

☒ The axis scales are clearly visible. Include numbers along axes only for bottom left plot of group (a 'group' is an analysis of identical markers).

☒ All plots are contour plots with outliers or pseudocolor plots.

☒ A numerical value for number of cells or percentage (with statistics) is provided.

Methodology

Sample preparation	see Methods, in the section "SMART-seq: Single cell cDNA synthesis and sequencing library generation"
Instrument	BD FACSAria Fusion

Software	BD FACSDiva Software
Cell population abundance	Population of sorted-tdTom positive cells in GlastCreERT; R26-tdTomato mouse retina was about 0.3%. The purity of sorted fraction was evaluated by qPCR.
Gating strategy	In every experiments, hoechst-unstained nuclei sample and tdTomato negative nuclei were used as negative controls to check the background signal. Multiplet cells were excluded by singlet gate and nucleus-containing cells (hoechst positive) were selected by Pacific blue channel. tdTom positive cells and negative cells from nucleus-containing cells were determined by PE channel.

☒ Tick this box to confirm that a figure exemplifying the gating strategy is provided in the Supplementary Information.
